# Single‐cell RNA sequencing reveals characteristics of myeloid cells in post-acute sequelae of SARS-CoV-2 patients with persistent respiratory symptoms

**DOI:** 10.3389/fimmu.2023.1268510

**Published:** 2024-01-08

**Authors:** Hyundong Yoon, Logan S. Dean, Boonyanudh Jiyarom, Vedbar S. Khadka, Youping Deng, Vivek R. Nerurkar, Dominic C. Chow, Cecilia M. Shikuma, Gehan Devendra, Youngil Koh, Juwon Park

**Affiliations:** ^1^ Cancer Research Institute, Seoul National University College of Medicine, Seoul, Republic of Korea; ^2^ Hawaii Center for AIDS, University of Hawai’i at Mānoa, Honolulu, HI, United States; ^3^ Tropical Medicine, Medical Microbiology, and Pharmacology, John A. Burns School Medicine, University of Hawai’i at Manoa, Honolulu, HI, United States; ^4^ Bioinformatics Core, Department of Quantitative Health Sciences, John A. Burns School of Medicine, University of Hawaii, Mānoa, Honolulu, HI, United States; ^5^ Department of Medicine, John A. Burns School of Medicine, University of Hawai’i at Mānoa, Honolulu, HI, United States; ^6^ Department of Pulmonary and Critical Care, Queen’s Medical Center, Honolulu, HI, United States; ^7^ Department of Internal Medicine, Seoul National University Hospital, Seoul, Republic of Korea

**Keywords:** SARS-CoV-2, long-COVID, post-acute sequalae of SARS-CoV-2 infection, pulmonary sequelae, single-cell RNA sequencing, monocyte

## Abstract

**Background:**

Although our understanding of the immunopathology and subsequent risk and severity of COVID-19 disease is evolving, a detailed account of immune responses that contribute to the long-term consequences of pulmonary complications in COVID-19 infection remains unclear. Few studies have detailed the immune and cytokine profiles associated with post-acute sequelae of SARS-CoV-2 infection (PASC) with persistent pulmonary symptoms. The dysregulation of the immune system that drives pulmonary sequelae in COVID-19 survivors and PASC sufferers remains largely unknown.

**Results:**

To characterize the immunological features of pulmonary PASC (PPASC), we performed droplet-based single-cell RNA sequencing (scRNA-seq) to study the transcriptomic profiles of peripheral blood mononuclear cells (PBMCs) from a participant naïve to SARS-CoV-2 (Control) (n=1) and infected with SARS-CoV-2 with chronic pulmonary symptoms (PPASC) (n=2). After integrating scRNA-seq data with a naïve participant from a published dataset, 11 distinct cell populations were identified based on the expression of canonical markers. The proportion of myeloid-lineage cells ([MLCs]; CD14^+^/CD16^+^monocytes, and dendritic cells) was increased in PPASC (n=2) compared to controls (n=2). MLCs from PPASC displayed up-regulation of genes associated with pulmonary symptoms/fibrosis, while glycolysis metabolism-related genes were downregulated. Similarly, pathway analysis showed that fibrosis-related (*VEGF*, *WNT*, and *SMAD*) and cell death pathways were up-regulated, but immune pathways were down-regulated in PPASC. Further comparison of PPASC with scRNA-seq data with Severe COVID-19 (n=4) data demonstrated enrichment of fibrotic transcriptional signatures. In PPASC, we observed interactive *VEGF* ligand-receptor pairs among MLCs, and network modules in CD14^+^ (cluster 4) and CD16^+^ (Cluster 5) monocytes displayed a significant enrichment for biological pathways linked to adverse COVID-19 outcomes, fibrosis, and angiogenesis. Further analysis revealed a distinct metabolic alteration in MLCs with a down-regulation of glycolysis/gluconeogenesis in PPASC compared to SARS-CoV-2 naïve samples.

**Conclusion:**

Analysis of a small scRNA-seq dataset demonstrated alterations in the immune response and cellular landscape in PPASC. The presence of elevated MLC levels and their corresponding gene signatures associated with fibrosis, immune response suppression, and altered metabolic states suggests a potential role in PPASC development.

## Background

Three years have passed since the outbreak of the novel coronavirus disease 19 [COVID-19], caused by severe acute respiratory syndrome virus 2 [SARS-CoV-2] (1). COVID-19 is a global public health crisis and has had profound impacts on economic growth and social structures (2–4). The advent of COVID-19 vaccines has effectively reduced the risk of SARS-CoV-2 infection and prevented death and severe illness following acute infection (5, 6). However, approximately 10-30% of COVID-19 survivors experience chronic health conditions (‘sequelae’) that persist longer than 3 months and develop within 28 days of acute illness (7–10). These ongoing health problems are termed Long COVID or Post-COVID Syndrome, but also referred to as “post-acute sequelae of SARS-CoV-2” (PASC). PASC symptoms reported by survivors vary, but common symptoms include fatigue, insomnia, dyspnea, and coughing (11). A subset of PASC continues to experience persistent pulmonary symptoms even after recovery from the acute infection (described hereafter as PPASC [pulmonary PASC]) (11). Recent large cohort studies demonstrated that COVID-19 survivors have a greater risk of developing comorbidities, particularly diabetes (12), as well as cardiovascular (13) and kidney (14) diseases. Considering the global prevalence of PASC cases (15–17) and the impact of sequelae on COVID-19 survivors’ quality of life and disability-adjusted life years (18–21), PASC continues to present a significant global burden to healthcare infrastructure.

The etiology of PASC, and therefore PPASC, is not yet known, but ongoing studies have provided risk factors and potential predictors of PASC development. Factors, such as female sex, age, comorbidities, smoking, and social determinants of health (low socioeconomic status and racial/ethnic minority) are associated with an increased risk of PASC development (22–24). While the severity of COVID-19 is suspected to be a risk factor for PASC (25), evidence continues to present mixed conclusions (26–32).

Investigations into the immunopathology associated with PASC have revealed that aberrant cellular and humoral immune responses are drivers of PASC (33–36). Comparison of the circulating proteome between individuals with acute COVID-19, COVID-19-recovered, and PASC demonstrates no significant differences in most cytokines related to inflammation, nor a disease-specific signature among the groups (33, 37). However, a consensus of studies has demonstrated that IL-6, TNF, and IL-1β are commonly elevated in individuals with PASC (38–40), suggesting a pro-inflammatory etiology.

Cellular metabolic dysregulation is one of the major features of SARS-CoV-2 infection and a key determinant of disease severity (41, 42). Furthermore, epidemiological observations demonstrated that individuals who had metabolic comorbidities, such as diabetes mellitus, hyperglycemia, or obesity, were at a significantly increased risk of severe COVID-19 (43). Lung epithelial cells and monocytes infected with SARS-CoV-2 demonstrate lipid dysregulation with excessive lipid droplet accumulation within the cells (44–46). Inhibition of lipid droplet biogenesis was demonstrated to inhibit the proinflammatory response and replication of SARS-CoV-2 within infected monocytes (44), suggesting a metabolic rearrangement toward lipid use for viral replication. Investigations into glycolysis metabolic pathways reveal a strong propensity for upregulation within macrophages, monocytes, and T cells, consistent with altered cell activation and attenuated T cells’ effector functionality (47, 48). Recent evidence demonstrates a metabolic shift toward a pro-resolution and remodeling phenotype in macrophages isolated from PASC patients (49), indicating changes in plasma metabolites after SARS-CoV-2 infection led to profound and prolonged cellular metabolic disruption and subsequent cell dysfunction.

The persistence of SARS-CoV-2 protein and RNA in both immune cells and tissues (50, 51) has arisen as potential factors for PASC development (52). The persistence of viral components is well documented to have prolonged impacts on immune cell functionality and polarization (53–55) and, therefore, likely influences PASC etiology. It is likely a combination of a multitude of the aforementioned factors driving PASC development. As such, each avenue of investigation remains a viable option for discovery, with the goal of predictive and/or therapeutic intervention innovations for PASC.

Recent single‐cell RNA sequencing (scRNA‐seq) data has demonstrated profound phenotypic alteration of immune cells in COVID-19 convalescents with persistent symptoms. Innate and adaptive immune cell perturbations are evident by 12 weeks post-infection and sustained within individuals who develop PASC (56). Another large-scale scRNA-seq endeavor revealed the predictive value of cortisol levels as well as circadian rhythm-related gene signatures as predictive of PASC development (57). Although ongoing PASC studies are focused on advancing our understanding of its' pathophysiology, the mechanisms underlying persistent pulmonary sequelae secondary to SARS-CoV-2 infection remain largely unexplored. The mechanism in which key immune cell subsets change their functionalities in PASC with sustained pulmonary symptoms has remained undefined. Therefore, the identification of primary cell types associated with PPASC and signaling pathways mediated by immune cell alteration and activation are crucial to obtaining insights into immune cell perturbation that contributes to prolonged pulmonary sequelae in PASC.

In this study, we analyzed Peripheral blood mononuclear cells (PBMC) PBMC from individuals who had developed PPASC after SARS-CoV-2 infection and uninfected controls. The immune profiling and transcriptomic data revealed alterations of myeloid lineage immune cells, notably monocytes and dendritic cells in PPASC. We further explored individual cell-cell interactions on immune cell types and performed metabolic analyses, demonstrating a profound association with fibrosis, vascularization, and characteristics of a reparative immune environment. Together, our results provide evidence that perturbations undergone within the myeloid lineage of immune cells are likely associated with ongoing pro-fibrotic processes that may therefore be contributing specifically to PPASC development. This provides valuable insight into the utility of targeting immune cell populations to ameliorate pulmonary sequelae among PPASC individuals.

## Methods

### Peripheral blood mononuclear cell isolation

Human venous peripheral blood samples were taken at the state’s main tertiary care hospital-Ambulatory Post-COVID Clinic (Queen’s Medical Center, Honolulu, Hawaii) and collected in ethylenediaminetetraacetic acid (EDTA) tubes (BD, Vacutainer) by venipuncture. In brief, venous blood was diluted with an equal volume of phosphate-buffered saline (PBS) and layered on top of Ficoll-Paque Plus (GE Healthcare Biosciences, Piscataway, NJ) following the manufacturer’s protocol. PBMC were separated by centrifugation at 400 × g for 30 minutes at room temperature (RT). PBMCs were collected from the buffy coat, red blood cells were lysed, and the remaining pellet was washed twice in PBS supplemented with 2% fetal bovine serum (FBS). Cells were then counted, viability determined, and cryopreserved at 5 million cells/vial. One participant naïve to SARS-CoV-2 infection and two participants with prolonged COVID-19 pulmonary symptoms, confirmed via pulmonary function tests (PFTs), were selected for single-cell sequencing analysis. This study was approved by the Queen’s Medical Center Research & Institutional Review Committee RA-2020-053 and by the University of Hawaii Institutional Review Board 2020-00406. Written informed consent was obtained from all participants and all assays were performed according to institutional guidelines and regulations.

### Pulmonary function tests

PFTs was performed on individuals with PPASC. All PPASC participants underwent PFTs (Vyaire) with the measurements of forced vital capacity (FVC), forced expiratory volume in 1 second (FEV1), total lung capacity (TLC), and diffusion capacity corrected for hemoglobin percent predicted (DLCOc%) interpreted in accordance with American Thoracic Society (ATS) guidelines (58).

### Fluorescence-assisted cell sorting

For live PBMC purification for scRNA-seq, PBMC were stained with BV711-CD45 (Clone H130, 1:200 dilution, Biolegend, San Diego, CA) for 30 minutes at RT after adding Human TruStain FcX (1:200 dilution, Biolegend, San Diego, CA) for 15 minutes. Propidium iodide (PI) was added just before cell acquisition to assess cell viability. UltraComp eBeads Compensation Beads (Thermo Fisher Scientific, 01-2222-42) were used for compensation. Approximately 98-99% of total PBMC were identified as live and CD45^+^ and were sorted into 1% bovine serum albumin (BSA) in DPBS by a FACSAria IIu Cell Sorter (BD Biosciences, Franklin Lakes, NJ). Sorted cell suspensions were pelleted at 350 x g for 5 minutes at 4°C, and cells were resuspended in 0.1% BSA in Dulbecco's Phosphate Buffered Saline (DPBS) to approximately 1,000 cells/μl.

### Single-cell RNA-sequencing

Cell concentration and viability were confirmed using an automated cell counter Countess II (Thermo Fisher Scientific) with 0.4% trypan blue solution and samples with viability > 70% were further processed. To obtain single-cell gel beads-in-emulsion (GEM), cell suspension was pelleted by 400 x g for 5 minutes at 4°C, and cells were resuspended at a concentration of 1,000 cells/μl in 0.1% BSA in DPBS. scRNA-seq libraries were prepared using Chromium Next GEM Single-Cell 5’ Reagent Kit (10x Genomics). Briefly, GEMs were generated in Chromium Controller by combining barcoded Single-Cell VDJ 5’ Gel Beads v1.1, a Master Mix with mixture of single cells, and Partitioning Oil on Chromium Next GEM Chip G. To achieve single-cell resolution, cells were delivered at a limiting dilution, such that the majority (~90-99%) of generated GEMs contains no cell, while the remainder largely contain a single cell.

Immediately following GEM generation, the Gel Beads were dissolved and any co-partitioned cell was lysed. Oligonucleotides containing (i) an Illumina R1 sequence (read 1 primer sequence), (ii) a 16 nucleotide (nt) 10x Barcode, (iii) a 10 nt unique molecular identifier (UMI), and (iv) 13 nt template switch oligo (TSO) were released and mixed with the cell lysate and a Master Mix containing reverse transcription (RT) reagents and poly(dT) RT primers, resulting in full-length cDNA from poly-adenylated mRNA. GEMs were broken and pooled after GEM-RT reaction mixtures were recovered. Silane magnetic beads were used to purify the 10x Barcoded first-strand cDNA from the post-GEM-RT reaction mixture, which includes leftover biochemical reagents and primers. After cleanup, 10x Barcoded, full-length cDNA was amplified via PCR with primers (Forward primer [P5]: 5’-AATGATACGGCGACCACCGAGA-3’ and Reverse primer [P7]: 5’-CAAGCAGAAGACGGCATACGAGAT-3’against common 5’ and 3’ ends added during GEM-RT). Amplification generated sufficient material to construct 5’ Gene Expression libraries. Enzymatic fragmentation and size selection were used to optimize the cDNA amplicon size prior to the 5’ Gene Expression library construction. P5, P7, a sample index, and Illumina R2 sequence (read 2 primer sequence) were added via End Repair, A-tailing, Adaptor Ligation, and Sample Index PCR. The final libraries contain the P5 and P7 priming sites used in Illumina sequencers. Library quality and size distribution were confirmed on Agilent Bioanalyzer High Sensitivity DNA chips (Agilent). Library quantification was performed with qPCR using the KAPA Library Quantification Kit for Illumina Platforms (KAPA/Roche). Libraries were normalized, denatured, and diluted to obtain 1.5 pM molarity. Libraries were sequenced on the NextSeq500 instrument at a depth of a minimum of 20,000 reads/cell using 26 x 91 bp read settings.

### scRNA data merge and integration analysis

The scRNA-seq raw fastq files were aligned to the human reference genome (ver. GRch38) using the Cell Ranger pipeline (59) to generate a raw gene-by-cell count matrix. To integrate scRNA data generated from different batches, Harmony (60) was used to mitigate batch effects by merging and integrating the data. We chose a publicly available scRNA-seq dataset (GSM4509024) (61) that contained SARS-CoV-2 naïve and Severe COVID-19 PBMC samples with the study participants of similar age to our own samples. Our scRNA-seq data of PBMC (PPASC; n=2, Control; n=1) or PPASC data were combined with a naïve participant and then severe COVID-19 patients (n=4). DropletUtils was utilized to assess deviations from the ambient cell profile and empty droplets were identified (62), and cells with an FDR < 0.01 were considered statistically significant and retained as “real cells,” while those with an false discovery rates (FDR) > 0.01 were deemed “empty droplets” and removed. The Scater package was used for the calculation of quality control (QC) metrics, visualized through principal component analysis (PCA) (63). A total of 34,139 cells passed quality control (QC) for the integrated data of PPASC and Controls. A total of 34,070 cells passed QC for the integrated data of PPASC and Severe (QC; Removing empty droplets, cells with low UMI/feature counts, high mitochondrial gene expression indicative of dying cells). Via these methods, 5,861 cells were removed (**Supplementary Figures 1**-**4**). The Seurat package’s merge function was used to combine individual samples and remove cell-specific biases. The scran package’s quickCluster function was utilized for grouping cells. Cell-specific size factors were calculated using the computeSumFactors function within scran. Raw counts of each cell were normalized by cell-specific size factor and log2-transformed with a pseudo-count of 1; highly variable genes (HVG) were defined based on the variance of the log expression profiles of each gene for decomposed technical and biological components by fitted mean-variance trends through the modelGeneVar function within scran. HVG were defined as those with FDR < 0.05. Downstream analysis involved computing 20 principal components (PCs), constructing a shared nearest neighbor (SNN) graph, and performing cell clustering using the Seurat package. Batch effects in the combined individual samples were addressed by applying 50 PCs and the RunHarmony function from the Harmony package. The detailed pipeline code is available on GitHub (https://github.com/lagom2728/PPASC-SARS-CoV-2-Paper-2023).

### Cell type identification

Cells were identified based on differential expressions of canonical marker genes and clustered accordingly (64). Additionally, cell types were confirmed by visual confirmation of the average expression values of canonical marker genes (64). Cell proportions were confirmed by generating cell type percentages, followed by a permutation test and bootstrapping to validate the statistically significant differences in proportions. As a result, cells were categorized into statistically significant groups based on an increase or decrease in the proportion of cells meeting the following criteria: relative differences in cell proportions for Log2 Fold Distribution (FD) in each group > 0.3, Log2FD < -0.3, and FDR < 0.05.

### Differentially expressed gene analysis

To assess the differential gene expression in the scRNA data of two groups (each PPASC and Control), we conducted differential expressed gene analysis with the built-in framework of MAST (65). In the analysis results, significant up- and down-regulated genes were classified based on statistical criteria of avg_log2FC > 0.25, < -0.25, and p-value < 0.05.

### Gene set enrichment analysis

To conduct the analysis, Hallmark gene sets (H), Curated gene sets (C2), and Ontology gene sets (C5) databases of MsigDB (66) were selectively used and analyzed (67). Significant regulated pathways were categorized by their normalized enrichment score (NES) as enriched (>0) or depleted (<0), and p-values < 0.05.

### Transcription factor enrichment analysis

The DoRothEA database (68) was used to identify transcription factors for differentially expressed target genes. The transcription factors from DoRothEA are complemented by an empirical measure of confidence, reflecting the certainty in their regulons. This confidence level is categorized into grades ranging from A (highest confidence) to E. In this particular benchmark, we specifically considered only the transcription factors (TFs) with confidence levels A and B, collectively referred to as DoRothEA (AB), used for transcription factor enrichment analysis.

TF enrichment analysis was performed using the msviper function within the viper package (69). Using the control patient as a reference, genes that were differentially expressed in each cell type via the Log2FD were identified and NES was calculated. TFs were classified as statistically significantly upregulated or downregulated according to the NES criteria and p-adjusted value thresholds (NES > 0, < 0, p-value < 0.05).

### Cell-to-cell interactions

The human ligand-receptor (only Secreted signaling) database was used to analyze the ligand-receptor interaction between cells (70). Cells expressing less than 5% of genes were excluded, and only interactions with statistical significance (p-value < 0.05) among autocrine or paracrine interactions were classified. To compare the interaction signals between the two groups, the communication probability value was calculated based on the geometric mean expression level.

### Pseudo-bulk differentially expressed gene analysis

To make scRNA into pseudo-bulk RNA, we aggregated transcript count tables of all single-cells per sample to verify possible false positives in single-cell differentially expressed genes (DEGs). Differential expression analysis was performed (71), and genes satisfying LogFC > 0.25 or < -0.25, along with a p-value < 0.05 were classified as up- or down-regulated genes, respectively.

### Single-cell correlation network analysis

Integrated scRNA data was utilized to calculate Pearson correlation values (72). Subsequently, correlation networks were created for specific cell types, and the identification and validation of differentially expressed network modules were accomplished through enrichment analysis (73).

### Metabolic analysis

For metabolic analysis, we utilized the pipeline provided by the Compass package (74), specifically employing Single-Cell Flux Estimation Analysis and Single-Cell Flux Balance Analysis. These analytical tools were used to estimate cell-specific metabolic flux to infer the metabolic states of immune cells. For analysis of metabolite communication, we employed MEBOCOST (67). This model estimates the relative abundance of metabolic products based on the expression of genes encoding metabolic reaction enzymes. It additionally identifies cell-to-cell communication of metabolites and sensors by gathering enzyme-related genes from the Human Metabolome Database (75) across different cells.

## Results

### Alteration of myeloid-lineage cells in individuals with PPASC

Circulating immune cell levels and their functional status hold promise as biomarkers for assessing the severity of COVID-19. Analysis of blood immune cells in COVID-19 convalescents has been used to provide insight into the long-term consequences of host immune responses after SARS-CoV-2 infection. To explore immune system alteration associated with persistent lung sequelae after recovery from acute illness of SARS-CoV-2, we selected two participants who had reduced DLCOc% (<80%) by PFT among individuals with PPASC. They had persistent pulmonary symptoms greater than 5 months after onset of SARS-CoV-2 infection, were hospitalized for COVID-19 disease, and were fully vaccinated against SARS-CoV-2 (**Supplementary Table 1**). PASC symptoms included shortness of breath, chest pain, joint pain, brain fog, and depression. As a comparator, an age-matched participant naïve to SARS-CoV-2 infection (Control) was selected and confirmed negative via a SARS-CoV-2 nucleocapsid antibody test (**Supplementary Table 1**). Live CD45^+^ cells from two PPASC patients and this SARS-CoV-2 naïve individual were subjected to scRNAseq analysis utilizing the 10X Genomics Chromium system which yielded an integrated object of 34,139 cells after QC.

**Figure 1:**
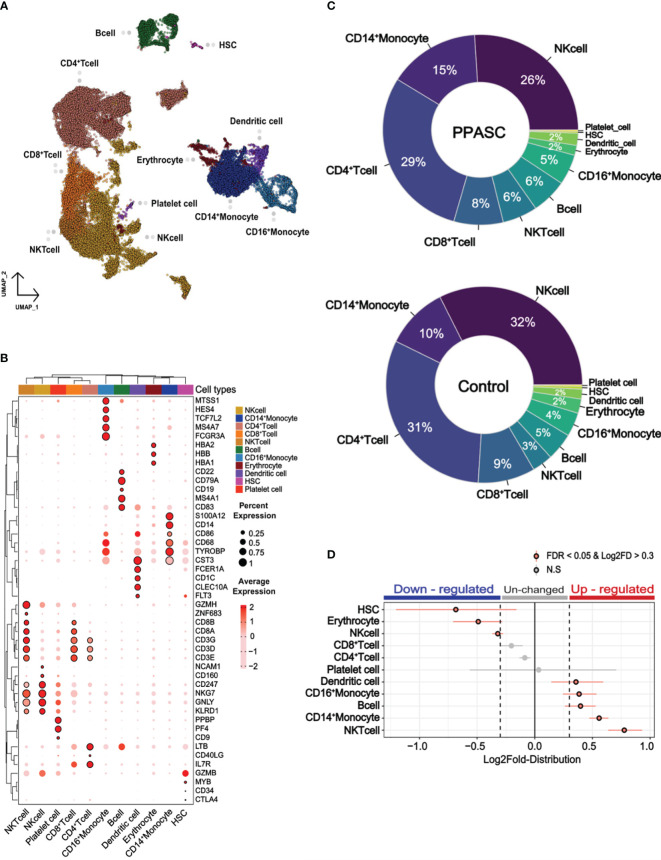
Cell type profile and differential cell type proportion analysis of PPASC and control participants. **(A)** scRNA data obtained from PBMCs with a total of 34,319 integrated cells within PPASC (n=2) and controls (n=2). The uniform manifold approximation and projection (UMAP) presentation of all merged samples and 11 cell types were clustered by gene signature. Each dot corresponds to a single cell and is colored according to cell type. **(B)** The annotation of clusters in the UMAP plot utilizing the expression of canonical marker genes from the Cell Marker Database 2.0. Dot plots present the average expression levels and the percentage of cells expressing each marker gene within the labeled cell types. The rows in the dot plots correspond to selected marker genes highly expressed in each cell cluster. **(C)** Cell type proportions for each cell type were manually calculated from samples of PPASC (n=2) and control (n=2) groups. The results were visualized through pie charts. **(D)** To detect statistical differences in cell proportions, a permutation test and bootstrapping approach were employed to compare the proportions of each cell type between the PPASC (n=2) and control (n=2) groups. The dot plot illustrates statistically significant relative differences in cell proportions, presenting the distribution of Log2FD in PPASC compared to controls.

We integrated our scRNA-seq data with a naïve participant from a public scRNA-seq dataset (GSM4509024) (61) to compare the transcriptome of PPASC (n=2) and controls (n=2). Each of the four independent scRNA-seq samples was subjected to uniform manifold approximation and projection (UMAP) based on RNA expression (**Figure 1A**). A total of 11 cell types were identified, including CD4^+^/CD8^+^ T cells, natural killer (NK) and natural killer T (NKT) cells, CD14^+^ and CD16^+^ monocytes, dendritic cells, platelets, hematopoietic stem cells (HSC), B cells, and erythrocytes (**Figure 1A**). Canonical marker genes of known cell types were enriched in each cluster and the marker expressions were used to annotate the clusters (**Figure 1B**). All identified immune cell populations were present in both SARS-CoV-2-infected and naïve participants (**Supplementary Figure 5**), albeit in differing proportions between PPASC and controls (**Figure 1C**). We further attempted to determine a vital feature for reflecting immune cell alterations associated with PPASC via the relative proportions of peripheral immune cells. We observed significant increases in myeloid cells (CD14^+^/CD16^+^ monocytes and dendritic cells), and NKT cells and B cells, whereas the proportions of NK cells, HSC, and erythrocytes were significantly decreased in PPASC compared to controls (**Figure 1D**).

### Identification of dysregulated genes and pathways related to PPASC

**Figure 2:**
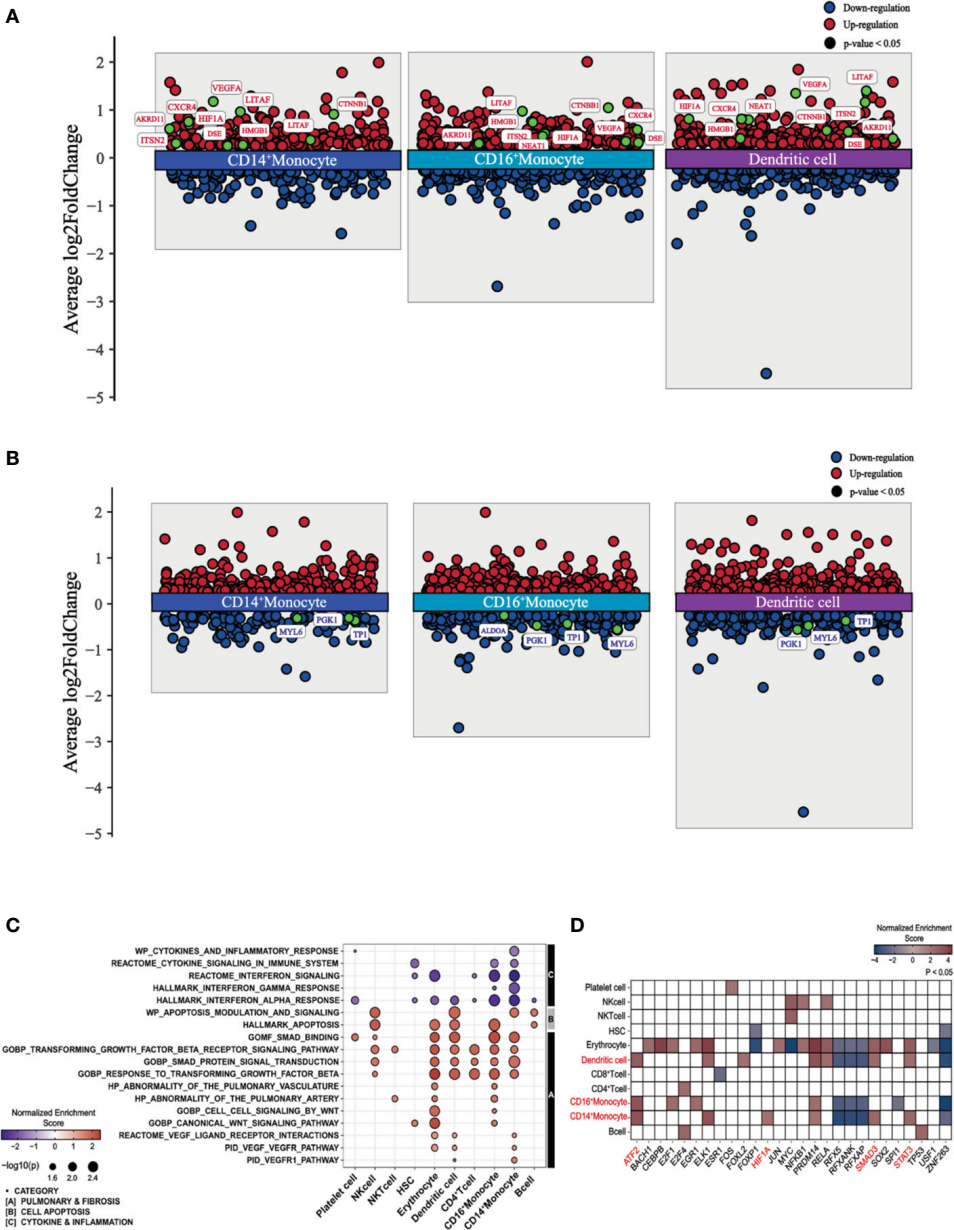
Differential gene expression, pathway enrichment, and transcription factor enrichment analysis of PPASC compared to controls. **(A)** Differentially expressed and upregulated genes with selective labeling of fibrosis-related genes in CD14^+^ and CD16^+^ monocytes and dendritic cell populations in PPASC (n=2) compared to controls (n=2), visualized as scatter plots. **(B)** Differentially expressed and downregulated genes with glycolysis-related genes in CD14^+^ and CD16^+^ monocytes and dendritic cell populations in PPASC compared to controls, visualized as scatter plots. Statistically significant up-regulated genes are illustrated with red dots and statistically significant down-regulated genes are illustrated with blue dots for both **(A, B)**. **(C)** Comparative analysis of functional pathways between PPASC and control groups. Gene set enriched pathways in PPASC compared to controls across all immune cell types visualized by dot plot and grouped by selective pathway category. Up-regulated pathways are represented by red dots and down-regulated pathways by blue dots **(D)** Inferred differentially enriched transcription factors in PPASC compared to control groups across all cell types based on DoRothEA’s target genes database, visualized as a heatmap (Red; up-regulation, Blue; down-regulation). Transcription factors related to fibrosis that were found consistently enriched in MLCs are highlighted in red.

To explore the characteristics of immune cells and identify the molecular changes associated with PPASC, we performed a detailed analysis of the DEGs of immune cells from PPASC compared with those from the controls. Our scRNA-seq analysis identified pronounced alterations in myeloid cells. Furthermore, we found that elevated circulating monocyte levels and their activation were observed in COVID-19 convalescents (76). Thus, we focused our attention on myeloid cells, particularly CD14^+^ and CD16^+^ monocytes, as well as dendritic cells, for downstream analysis hereafter referred to as myeloid-lineage cells (MLCs). We found a total of 872 DEGs in monocytes and dendritic cells: CD14^+^ monocytes (228 upregulated and 132 downregulated), CD16^+^ monocytes (303 upregulated and 226 downregulated), and dendritic cells (332 upregulated and 217 downregulated) (**Supplementary Figures 6A, B**). In addition, we demonstrated shared DEGs across three cell types (118 upregulated and 54 downregulated) (**Supplementary Figures 6A, B**). Interestingly, our data showed that genes known to be associated with pulmonary fibrosis (*ANKRD11, CTNNB1, CXCR4, HIF1A, HMGB1, ITSN2, LITAF, NEAT1, VEGFA*, and *DSE*) (77–84) were upregulated in MLCs. However, genes associated with glycolytic metabolism (*ALDOA, PGK1, TPI1*, and *MYL6*) were downregulated in these cell populations (**Figures 2A, B**).

Next, we performed gene set enrichment analysis (GSEA) across each cell type and conducted direct comparisons of the PPASC versus the control groups, respectively. Overall, compared to controls, PPASC displayed upregulated pathways related to pulmonary fibrosis, such as *VEGF*, *WNT*, and *TGF-β* and cell apoptosis, while pathways related to cytokine/inflammation (including interferon) were downregulated in MLCs (**Figure 2C**). The *VEGF* pathway was upregulated in CD14^+^ monocytes and dendritic cells, while the *WNT* pathway was upregulated only in CD16^+^ monocytes (**Figure 2C**).

To identify potential direct targets involved in the induction of profibrotic features in myeloid cells, we performed transcription factor enrichment analysis (TFEA) to identify transcription factors inferred from the perturbation of gene signatures between PPASC and controls. We found that a total of 27 transcription factors were significantly dysregulated in individual cell types between groups (**Figure 2D**). Consistent with earlier analyses, we found that the downstream transcription factors of *VEGFR* (*HIF1A*; CD14^+^ monocytes) and *TGFBR* (*SMAD* and *STAT3*; CD14^+^ monocytes and dendritic cells, ATF2; CD14^+^/CD16^+^ monocytes and dendritic cells) signaling were upregulated in MLCs (**Figure 2D**). These observations demonstrate that PPASC display enriched genes driving pulmonary symptoms and profibrotic features in MLCs and that increased MLC proportions and the altered gene signatures likely contribute to development of pulmonary sequelae after SARS-CoV-2 infection.

### Fibrotic transcriptional signatures are significantly enriched in MLCs from PPASC

We were intrigued as to how our findings in PPASC patients, who had recovered from acute COVID-19 disease and suffered ongoing symptoms, compared with patients who suffered severe COVID-19. We integrated a publicly available scRNA-seq dataset from severe COVID-19 patients, hereafter referred to as severe COVID-19 (n=4) (**Supplementary Figures 7A-F**) and observed 10 of the previously identified cell types via UMAP visualization of the combined dataset; however, HSCs were unidentifiable (**Supplementary Figures 8A, B**). The cell proportion makeup differed drastically between PPASC and severe COVID-19, with CD16^+^ monocytes and dendritic cells found to be significantly increased and CD14^+^ monocytes significantly decreased in PPASC compared to severe COVID-19 (**Supplementary Figures 8C, D**). MLCs demonstrated an up-regulation of fibrosis-associated genes, such as *VEGFA* and *HIF1A* in PPASC compared to severe COVID-19 (**Supplementary Figure 9A**). However, genes involved in glycolytic metabolism were not found to be downregulated in PPASC, compared to severe COVID-19 (**Supplementary Figure 9B**). Pulmonary fibrosis-related pathways and cell apoptosis and stress-related pathways were found to be upregulated in PPASC compared to severe MLCs (**Supplementary Figure 9C**). Immune response pathways were found to be downregulated in CD14^+^ monocytes but not in CD16^+^ monocytes or dendritic cells in PPASC compared to severe COVID-19 (**Supplementary Figure 9C**). TF enrichment analysis revealed upregulation of fibrotic-associated *ATF2*, *STAT3*, and *SMAD* family TFs across MLCs in PPASC compared to severe COVID-19. STAT3 was specifically upregulated in CD16^+^ monocytes, while ATF2 was upregulated in CD14^+^ monocytes (**Supplementary Figure 9D**). DEGs in MLCs of PPASC compared to the severe COVID-19 demonstrated a larger overlapping proportion of up-regulated genes (**Supplementary Figure 10A**). Few overlapping downregulated DEGs were noted in MLC populations (**Supplementary Figure 10B**) and were not inclusive of glycolytic genes as found in PPASC vs control MLCs (Data not shown). Specifically, via analysis of cell-to-cell interactions in MLCs, we found that VEGF-VEGFR interaction was only found in PPASC but not in severe COVID-19 (**Supplementary Figure 11A**). VEGFA and its ligand expression was minimal in CD14^+^ and CD16^+^ monocytes in the severe COVID-19 but was over 40% in dendritic cells (**Supplementary Figure 11B**). Pseudo-bulk DEG revealed no differential expression of VEGF in CD16^+^ monocytes but confirmed up-regulation of VEGF in CD14^+^ monocytes and dendritic cells in PPASC (**Supplementary Figure 11C**). These data suggest that VEGF expression patterns in MLCs clearly distinguished PPASC from severe COVID-19.

### Gene modules predict variable expressions and functionalities of monocytes in PPASC

**Figure 3:**
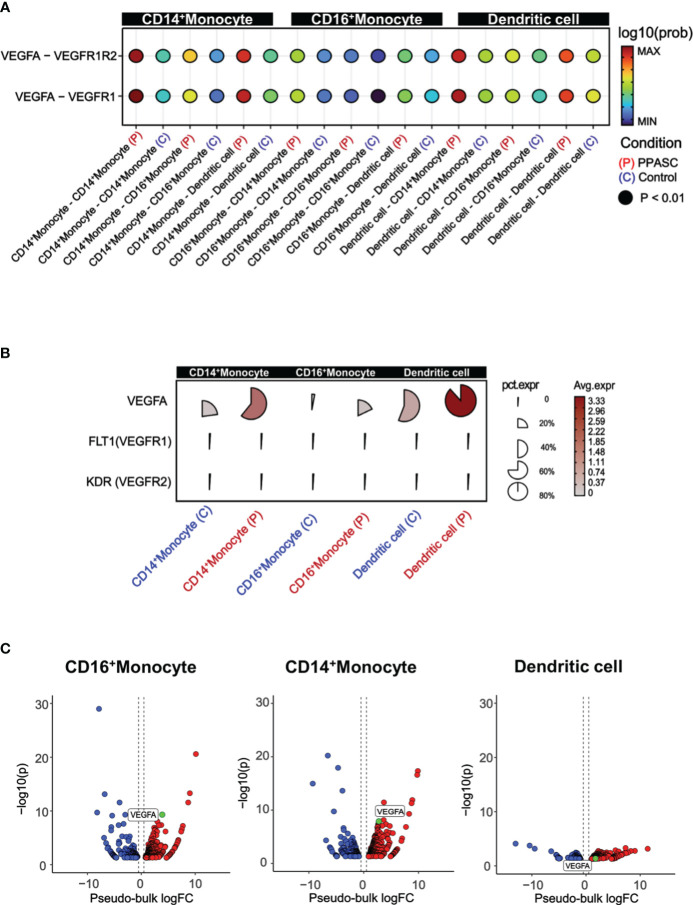
VEGF signaling, cell to cell interactions, and pathway enrichment analysis in PPASC compared to controls. **(A)** Geometric expression in cell-cell ligand-receptor interactions and VEGF signaling interactions were calculated between CD14^+^ and CD16^+^ monocytes and dendritic cells in PPASC (n=2) and control (n=2) groups. Statistically significant and comparable cell-cell interaction geometric expressions are shown on the x-axis, with ‘P’ and ‘C’ denoting the PPASC and control groups, respectively. **(B)** Single-cell details include average and percent expression of VEGF ligand-receptor genes in CD14^+^ and CD16^+^ monocytes and dendritic cells in PPASC and control groups. A high expression is denoted by red, and proximity to a circular form signifies a high percent expression in the cell type cluster. **(C)** For validation, aggregated single-cell expression data were processed as pseudo-bulk, and differentially expressed tests were conducted on VEGF ligand genes in CD14^+^ and CD16^+^ monocytes and dendritic cells in PPASC compared to control groups (Red; up-regulation, Blue; down-regulation).

To gain a comprehensive view of how the inferred targets may interact regarding PPASC development, we then identified potential cell–cell interactions and differential ligand-receptor interactions that are conserved in PPASC. The pathway of *VEGF* interaction (*VEGFA-FLT1/KDR*) was increased among MLCs, compared to controls (**Figure 3A**). We observed high-fidelity communications via *VEGF* pathways in MLCs via an autocrine or paracrine manner (**Figure 3A**). Furthermore, using pseudo-bulk single-cell data, we validated the expression of genes identified from ligand-receptor interaction analysis. *VEGFA* percent expression was increased in MLCs from PPASC compared to those cells from controls (**Figure 3B**). Notably, *VEGFA* was highly expressed in CD14^+^ and CD16^+^ monocytes, as well as dendritic cells (**Figures 3B, C**).

**Figure 4:**
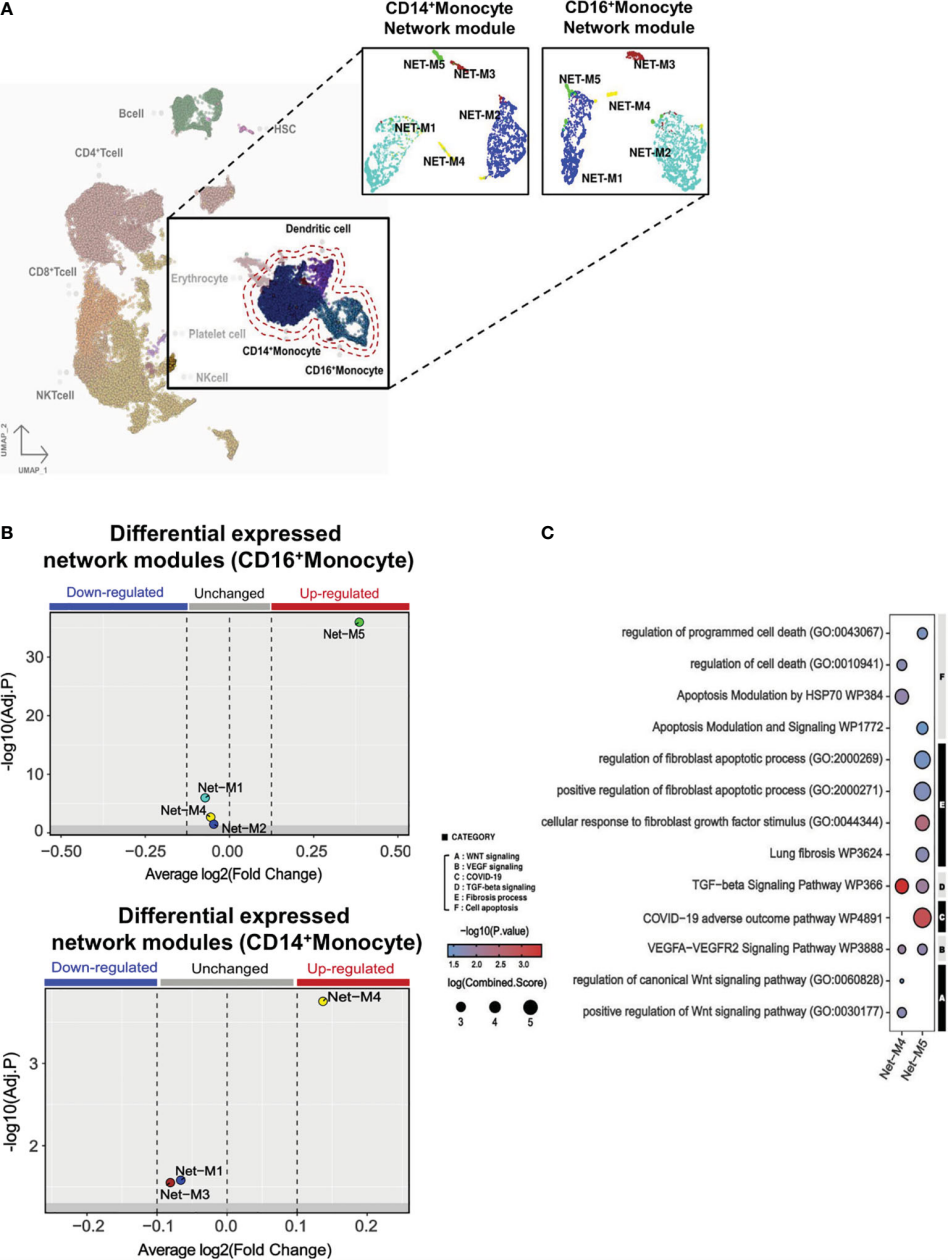
Monocyte gene modules in PPASC group suggest a pro-fibrotic skewing in CD14^+^ monocytes. **(A)** Gene correlation networks were constructed for CD14^+^ and CD16^+^ monocytes in PPASC (n=2) and control (n=2) groups. Cells were clustered into network modules using the UMAP clustering dimension reduction method in each correlation network. Each network module was formatted into five clusters. **(B)** Differential expression tests were performed using the Wilcoxon statistical framework in each network module for CD14^+^ and CD16^+^ monocytes in the PPASC and control groups. **(C)** Enriched pathway tests were conducted in the differentially expressed network modules 4 and 5. The results were visualized as dot plots, with selective pathway categories highlighted as functionally enriched pathways. Red dots signify up-regulation and blue dots signify down-regulation, with the size of the dot demonstrating the log fold change of the combined score.

Within the monocyte clusters (CD14^+^ and CD16^+^ monocytes), we further sub-grouped the signatures into gene correlation based on network modules (**Figure 4A**). Within CD14^+^ monocyte populations, network module 5 (Net-M5) was specifically found to be upregulated, while CD16^+^ monocyte demonstrated an upregulation of network module 4 (Net-M4) (**Figure 4B**). To identify the organizing hub genes in Net-M4 and M5, the top 60 genes with high eigengene-based connectivity (kME) values were selected (**Supplementary Figure 12**). In the Net-M4, genes involved in *VEGFA-VEGFR2* signaling (*NR4A1, TPM3, FAM120A*, and *NUMB*) and *WNT* signaling (*HHEX* and *APC*) were identified. The Net-M5 detected genes involved in lung fibrosis (*CXCL8* and *IL1B*), *VEGFA-VEGFR2* signaling (*NR4A1* and *CBL*), and *TGF-β* signaling (*JUNB* and *ATF3*) (**Supplementary Figure 7**). Furthermore, the pathway enrichment analysis of Net-M4 and Net-M5 revealed that the CD16^+^ M4 module was associated with *WNT*, *VEGF*, and *TGF-β*-signaling pathways (**Figure 4C**). The CD14^+^ M5 module was associated with COVID-19 adverse outcome linked pathways as well as fibroblast growth factor stimulation pathways. Interestingly, this same module in CD14^+^ monocytes revealed increases in pathways for fibroblast-specific apoptosis and general apoptotic pathways (**Figure 4C**).

### PPASC display transcriptome alterations indicative of metabolic perturbations in myeloid subsets

**Figure 5:**
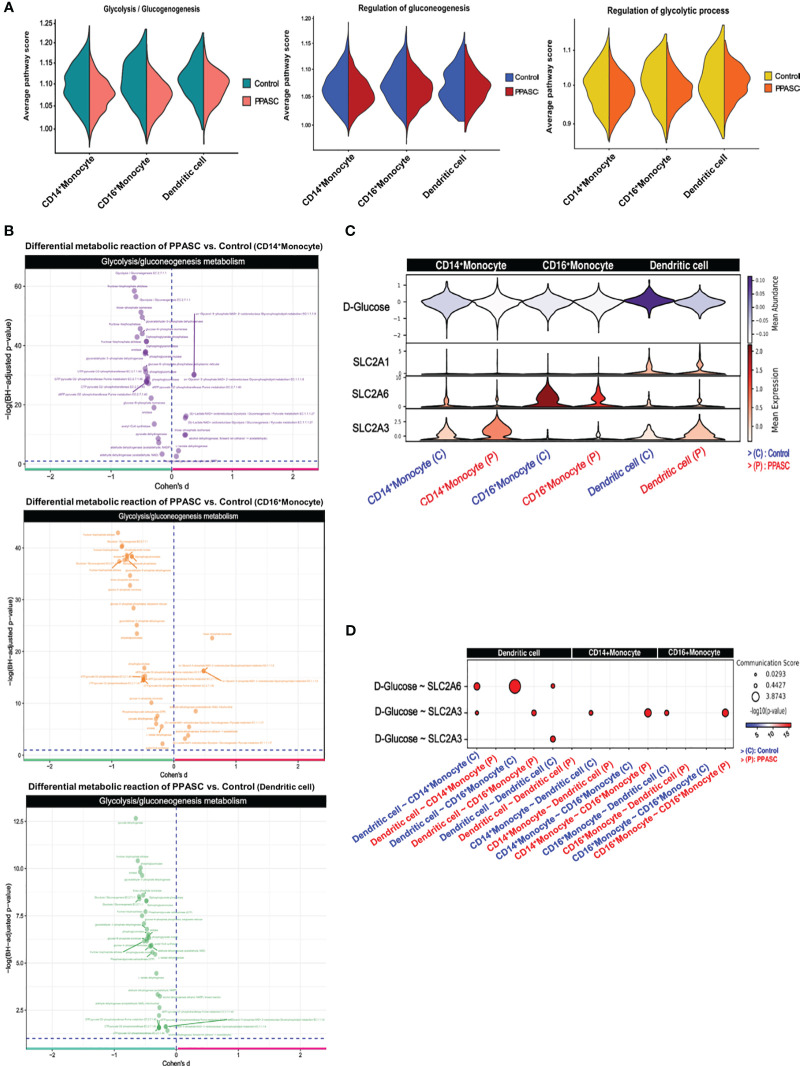
Metabolomic gene signatures in MLCs in Two PPASC individuals. **(A)** Gene set variation analysis (GSVA) was conducted using pathway signature gene sets to score average pathway scores in each cell. Glycolysis-related pathways were selectively examined in CD14^+^ and CD16^+^ monocytes and dendritic cells in PPASC (n=2) compared to control (n=2) groups. The results were split for each group and visualized as violin plots. **(B)** Measurement of differential metabolic reactions in glycolysis was performed using Cohen’s D statistical framework in CD14^+^ and CD16^+^ monocytes and dendritic cells. The results are visualized as a volcano plot with each dot representing a reaction in glycolysis metabolism. **(C)** Expression and abundance in glycolysis metabolite interaction were measured and visualized as a violin plot, depicting metabolite abundance and sensor gene expression between MLCs. **(D)** Inferred cell-to-cell glycolysis metabolite interactions in MLCs were scored and each communication between cells was visualized as a dot plot.

Reports of the metabolic impacts of SARS-CoV-2 infection and association with chronic fatigue and diabetic-like events during Long-COVID are well documented (85). These observations aligned with our findings of downregulated genes associated with glycolytic metabolism (**Figure 2B**) and prompted us to determine the impact of PASC on immunometabolism in individuals with persistent pulmonary sequelae. Using Gene Set Variation Analysis (GSVA) based on non-parametric unsupervised learning to infer the variability of gene set enrichment related to gluconeogenesis/glycolysis, we observed that the average score of glycolysis-related pathways in MLC populations was lower in PPASC than in controls (**Figure 5A**). The inferred metabolic state of individual cells using a database of integrated metabolic networks and metabolic flux balance analysis demonstrated that MLCs of PPASC exhibited downregulation of gluconeogenesis/glycolysis metabolic reactions compared to the control group (**Figure 5B**). Furthermore, inference of intercellular metabolite communication based on estimated metabolite scores and averaged sensor gene expression values by cell group demonstrated that the metabolite, D-Glucose, was directly associated with gluconeogenesis/glycolysis metabolism. Furthermore, these inferred interaction scores revealed decreased mean abundance values in MLCs of PPASC compared to the control group (**Figure 5C**). The calculated communication scores for metabolite-sensor partners revealed statistically significant interaction scores in MLCs of the control group but not in PPASC (**Figure 5D**). This suggests a lack of gluconeogenesis/glycolysis-related metabolite interaction or a diminished interaction in PPASC. In summary, these metabolic analysis observations suggest a decreased regulation of glucose-specific metabolic pathways in MLCs of PPASC.

## Discussion

Long-term perturbation of immune regulation and altered cytokine networks have been identified in COVID-19 convalescents and individuals with PASC tended to show more dysregulation of the immune system (34, 56). This evidence supports the hypothesis that an altered immune system after recovery against SARS-CoV-2 infection contributes to the development of PASC. While the epidemiological and clinical studies of pulmonary sequelae among PASC individuals are relatively advanced, mechanistic insights into the pathophysiological underpinnings of this condition are still limited. In this study, we performed scRNA-seq of the PBMCs from two PPASC patients and a naïve control with the inclusion of an integrated analysis with a published scRNA-seq control dataset (61). We revealed that the enrichment of myeloid-specific cellular subsets, CD14^+^ and CD16^+^ monocytes, and dendritic cells had increased profibrotic signatures in PPASC. We further explored cellular metabolism by analyzing metabolic reactions, metabolic pathway scoring, and cell-cell metabolite interactions from scRNA-seq data. We also compared the scRNA-seq data from our PPASC patients to a publicly available scRNA-seq dataset from PBMC obtained from patients who experienced severe COVID-19. To the best of our knowledge, this case study provides the first evidence for metabolic alterations in MLCs with a skewing toward decreases in gene expression of glycolysis and glucose utilization in PASC individuals with persistent pulmonary symptoms.

We recently reported persisting elevation of circulating monocytes in COVID-19 convalescents and cell activation was negatively correlated with lung function in individuals with PPASC (76). Consistent with this finding, our scRNA-seq data demonstrate continued dysregulation of the peripheral immune system, particularly MLCs, months after acute infection. Ryan et al. reported alterations of the blood transcriptome at 12-, 16-, and 24 weeks post-infection with SARS-CoV-2 when compared to naïve controls (56). Indeed, transcriptional dysregulation persisted in individuals with PASC compared to those who had completely recovered, until at least 24 weeks. Immunophenotyping results of PBMC demonstrated that specific subsets of CXCR3^+^ monocytes were increased in COVID-19 convalescents, but total monocytes were comparable between groups (56). None of the participants who were referred to the long-COVID clinic underwent PFTs nor were they classified as pulmonary long-COVID. The heterogeneity in PASC symptoms mirrors the existence of different pathological subgroups, suggesting that increased monocytic populations observed within our study may be in relation to pulmonary-specific immunopathology. In addition, PASC individuals with persistent pulmonary sequelae may have a selective immunological response that differs from the immunological response generated for other sequelae. Thus, more studies are essential for a better understanding of the pathophysiologic mechanisms underlying diverse spectrums of PASC symptoms.

An evolving body of evidence demonstrates heightened levels of plasma monocyte/macrophage‐related markers associated with proinflammation and fibrosis in COVID-19 convalescents (86). In concordance with our observed upregulation of gene expression related to pulmonary symptoms/fibrosis, these cell types could engage in sustaining the fibrotic process that may fuel pulmonary sequelae. Findings of fibrosing phenotypes in the lungs of post-COVID-19 patients have been demonstrated via histopathologic analysis (86). Examination of the lungs after 7 days of hospitalization revealed an increase in VEGFA and fibrotic pathology akin to organizing pneumonia (87). Interestingly, we observed upregulation of VEGF specifically in MLCs in PPASC compared to controls, but the patterns of up-regulation of VEGFA were more obvious when compared with severe COVID-19. CD14^+^ monocytes are known to differentiate into pro-fibrotic, fibroblast-like cells known as fibrocytes (88). Fibrocytes are known to migrate via CXCR4-CXCL12-mediated activity during fibrotic remodeling (89), and we found that this receptor was significantly upregulated in CD14^+^ monocyte populations in PPASC but not in severe COVID-19. TGF-β-signaling pathways are canonically known to skew and activate these CD14^+^-differentiated cells, and upregulation of TGF-β was also observed in MLCs from PPASC. As both TGF-β and VEGF have been touted as targets to inhibit lung fibrotic development and fibrotic processes systemically (90, 91), our findings suggest that circulating CD14^+^ cells from PPASC preferentially acquire profibrotic features.

Interestingly, we also found that CD16^+^ gene module interaction networks showed interactions with antigen-presentation genes. Antigen presentation on different monocyte subsets is well documented, with CD16^+^ intermediate and non-classical monocytes being the most prominent antigen presentation-capable monocytes (92). In line with their findings, CD16^+^ monocytes were found to increase their expression of MHC molecules, further promoted by stimulation of Th1 skewing cytokines. We did not observe upregulation of any Th1 or Th2 skewing cytokines, suggesting that this process is contributing toward our observed upregulation of MHC-2 molecules.

A recent study of plasma metabolomic profiles revealed dysregulation of metabolites with the enrichment of pathways for fatty acid metabolism and TCA cycles and high levels of fatty acid metabolites (93). Advances in the field of immunometabolism have demonstrated strong associations of specific metabolic pathways with cellular phenotype. In addition, the metabolic and metabolite level of investigation within a cell is considered the most accurate thermometer for cellular function as no processing or degradation (RNA/protein) is yet to occur. Although common residual symptoms of their study cohort were fatigue and brain fog, we observed similar trends of cellular metabolic alteration, displaying the downregulation of gluconeogenesis/glycolysis pathways in MLCs. Furthermore, in the CD16^+^ M4 module genes, suggesting a non-glycolysis-related metabolic signature, like ACSL4, a long-chain fatty acid ligase utilized for fatty acid beta-oxidation as well as OSBPL8, an oxysterol-binding protein for intracellular lipid transport, were identified. These results suggest that the downregulation of gluconeogenesis/glycolysis pathways appeared to be associated with a compensatory upregulation of genes associated with fatty acid oxidation and amino acid-based metabolism. However, we did not observe any DEGs related to metabolism between our PPASC and the severe COVID-19 groups. Future in-depth experimental work is required to understand whether metabolic reprogramming of monocytes after SARS-CoV-2 infection favors them to use fatty acid metabolism. Interestingly, the mean abundance of ATP was relatively comparable between MLCs in both PPASC and controls, suggesting that alternative metabolic pathways are pivotally compensatory in MLCs from PPASC, given the similar ATP production across the cell types.

A comprehensive review of PASC, particularly chronic fatigue syndrome, corroborates many of the findings observed from metabolic analyses of scRNA-seq in our study (27). Rewiring of cellular metabolism is directly linked to cellular function. Within monocyte populations, upregulation of glycolysis and glucose usage correlates with activation and differentiation toward an M1, i.e., pro-inflammatory monocyte/macrophage phenotype and functionality (94). This phenotype is defined by its pro-inflammatory cytokine production, such as IFN-γ, and stimulation, while M2 relates to an anti-inflammatory and regulatory role (95). The use of fatty acid pathways and decreased glycolytic reliance suggests pro-resolution (M2)-like phenotype and functionality within these monocytes. In the future, it will be necessary to elucidate the role of monocyte immunometabolism in the context of PPASC and whether metabolic dysfunction is a predisposing risk factor for PPASC.

Our study was limited by a small sample size. One of our control participants was from a matched, publicly available single-cell sequencing dataset. The comparator scRNA-seq data were further validated with a public dataset of other SARS-CoV-2 naïve single-cell sequencing data to ensure continuity. Although we utilized Harmony’s basic pipeline to mitigate batch effects, this small sample size predisposes variations within our data to have larger impacts that an increased sample size would mitigate. The inclusion of individuals who have fully recovered from COVID-19 would be informative to compare transcriptional changes of immune cells in peripheral blood in COVID-19 convalescents with/without residual symptoms. Also, this data would further identify MLC signatures specific to PASC with pulmonary sequelae. Further studies in large-scale cohort studies, specifically targeting the pulmonary sequelae of long-COVID and recovered survivors, are needed for data validation and enhancing our understanding of PPASC immunopathology.

## Conclusions

In conclusion, our analysis of scRNA-seq data reflecting persistent pulmonary sequelae among PASC has revealed alterations of peripheral blood cells and associated gene signatures. Collectively, our case study data demonstrate altered MLCs with specific skewing toward a pro-fibrotic and long-lived metabolic profile in PPASC. Such results will enable new insights into ongoing pulmonary sequelae among PASC individuals and also provide information into mechanisms underlying the immunopathology linked to pulmonary sequelae of PASC.

## Data availability statement

The datasets presented in this study can be found in online repositories. The names of the repository/repositories and accession number(s) can be found below: GSE235938 (GEO).

## Ethics statement

The studies involving humans were approved by Queen’s Medical Center Research and Institutional Review Committee (RA-2020-053) and University of Hawaii Institutional Review Board (2020-00406). The studies were conducted in accordance with the local legislation and institutional requirements. The participants provided their written informed consent to participate in this study.

## Author contributions

HY: Writing – original draft, Writing – review & editing, Data curation, Formal analysis, Methodology. LD: Formal analysis, Writing – original draft, Writing – review & editing. BJ: Data curation, Methodology, Writing – review & editing, Resources. VK: Data curation, Methodology, Writing – review & editing, Formal analysis, Validation. YD: Methodology, Validation, Writing – review & editing. VN: Writing – review & editing, Resources, Funding acquisition. DC: Writing – review & editing. CS: Writing – review & editing, Funding acquisition, Resources. GD: Writing – review & editing, Data curation, Funding acquisition. YK: Writing – review & editing. JP: Conceptualization, Funding acquisition, Supervision, Writing – original draft, Writing – review & editing.
