# Supplementary figures and images for: Single‐cell RNA sequencing reveals characteristics of myeloid cells in post-acute sequelae of SARS-CoV-2 patients with persistent respiratory symptoms
Source: Front Immunol. 2024 Jan 8;14:1268510. doi: 10.3389/fimmu.2023.1268510 (PMC10800799; doi:10.3389/fimmu.2023.1268510)

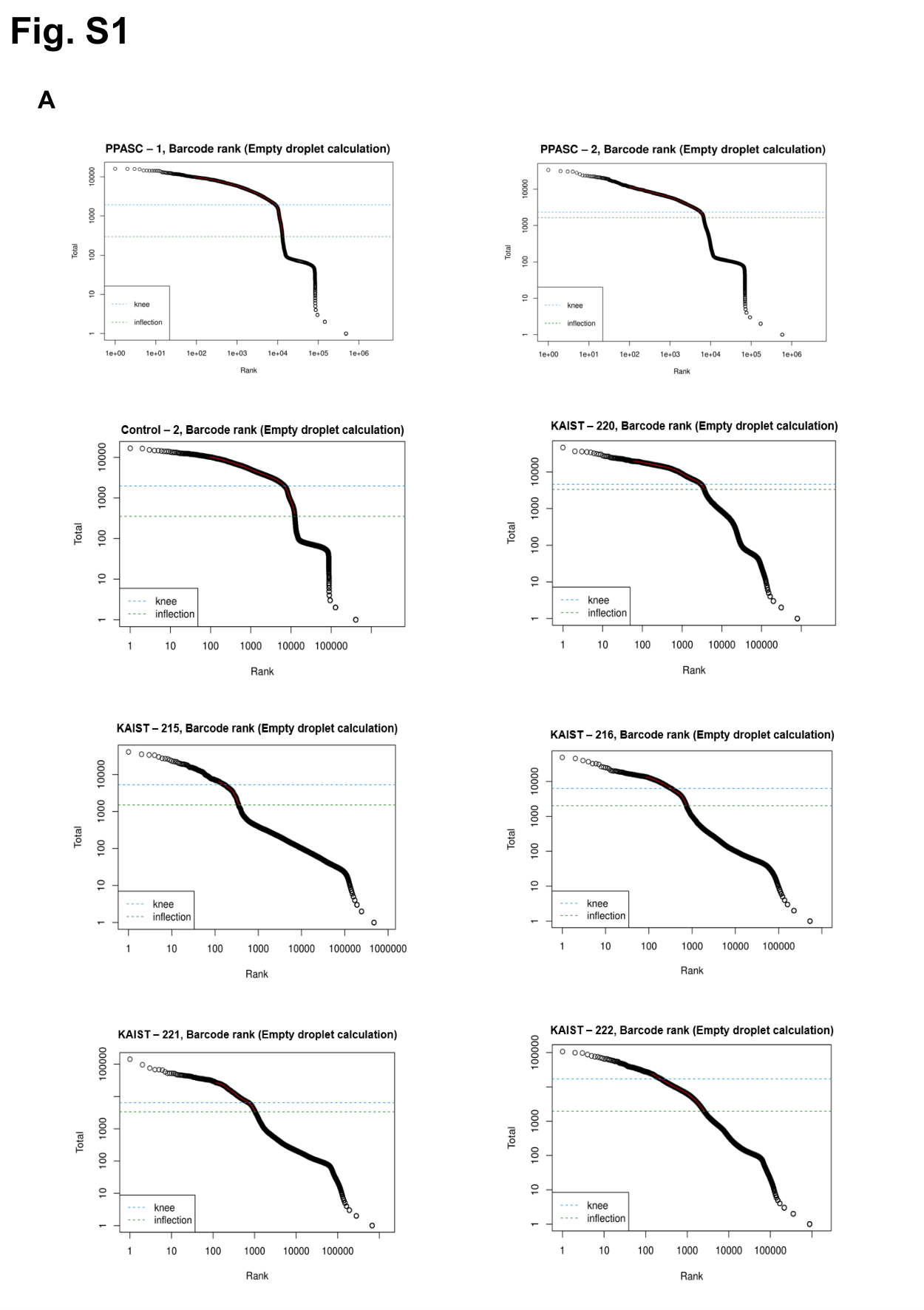

Supplement: Supplementary file 1 [file Image_1.tiff]

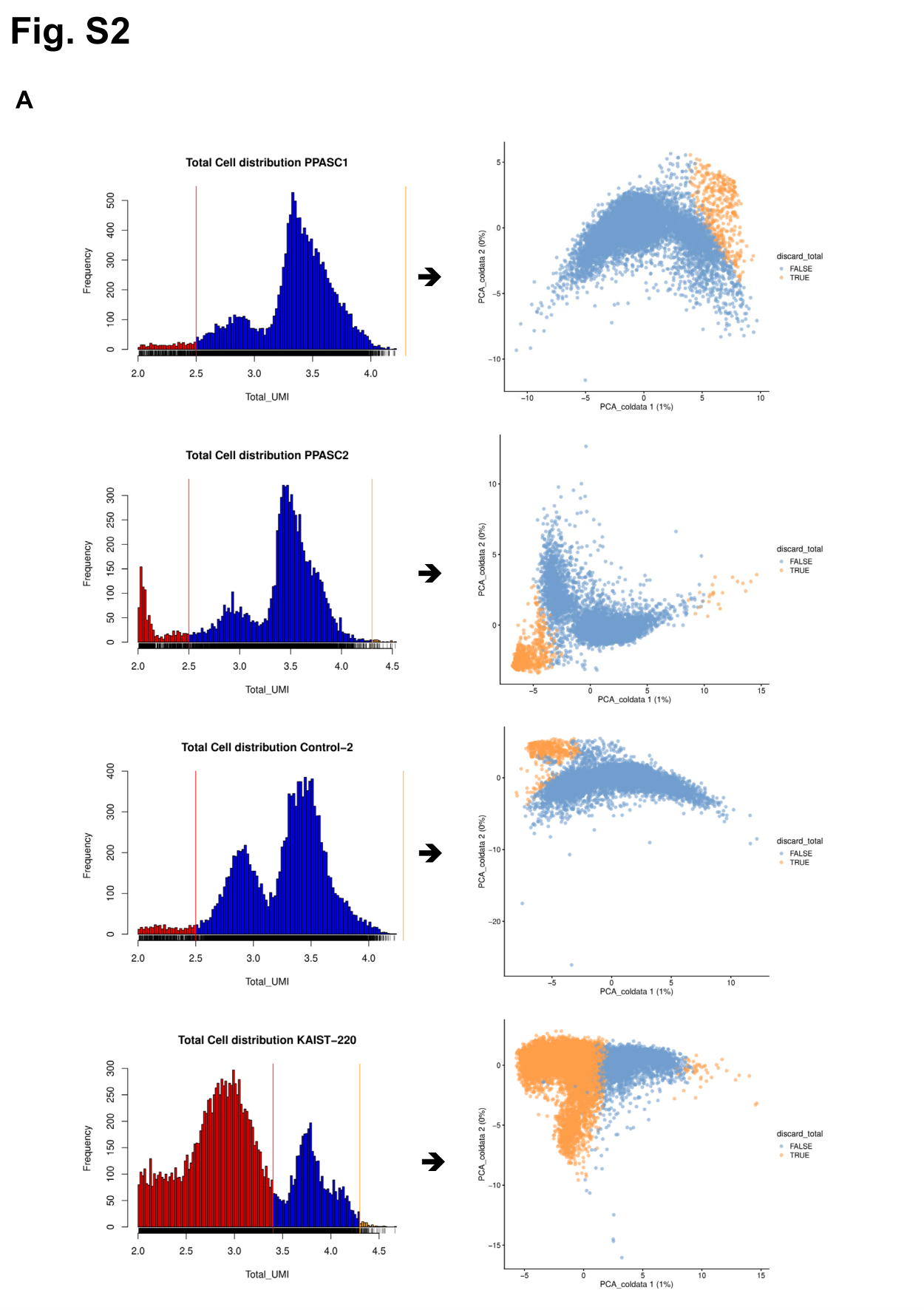

Supplement: Supplementary file 2 [file Image_2.tiff]

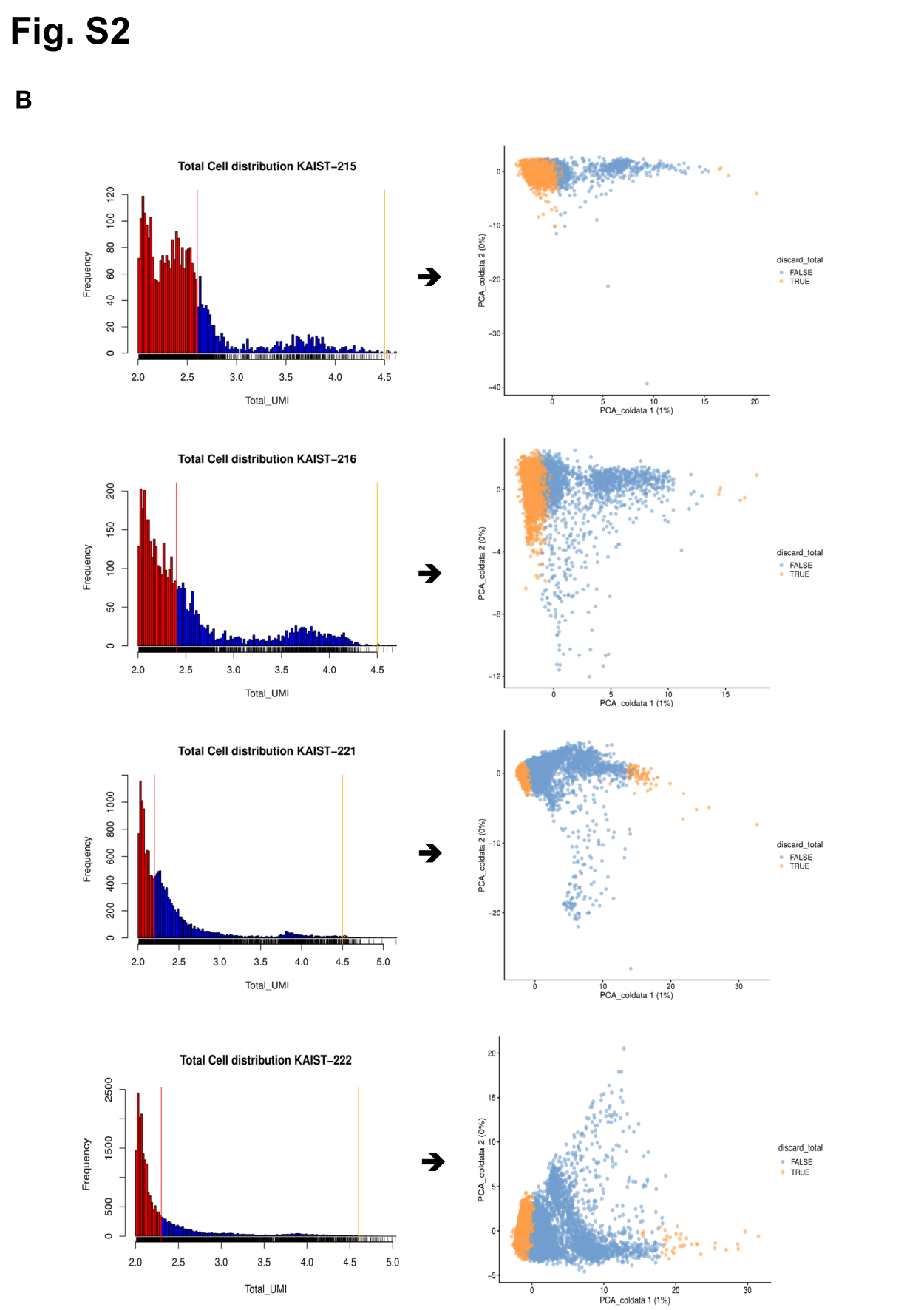

Supplement: Supplementary file 3 [file Image_3.tiff]

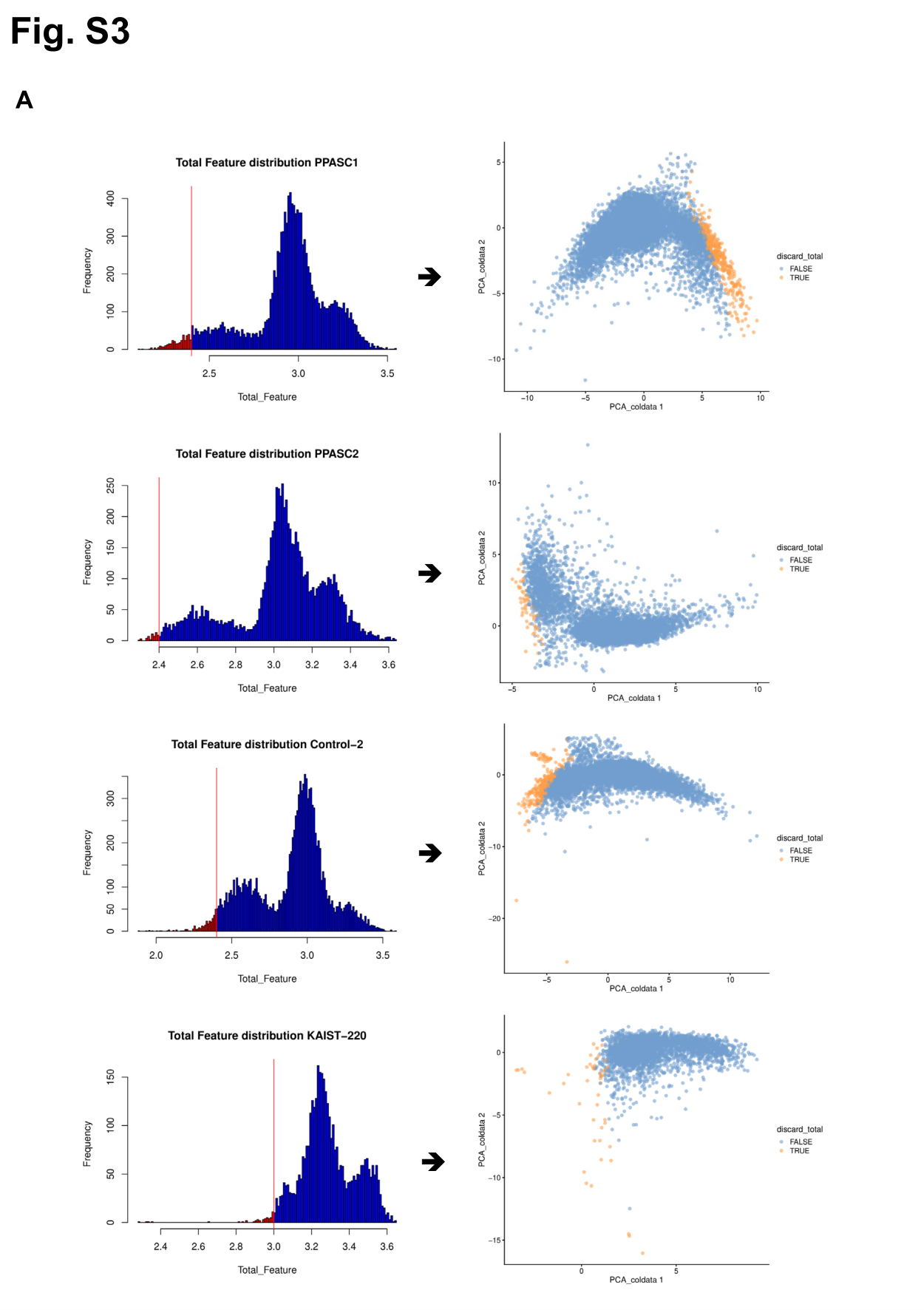

Supplement: Supplementary file 4 [file Image_4.tiff]

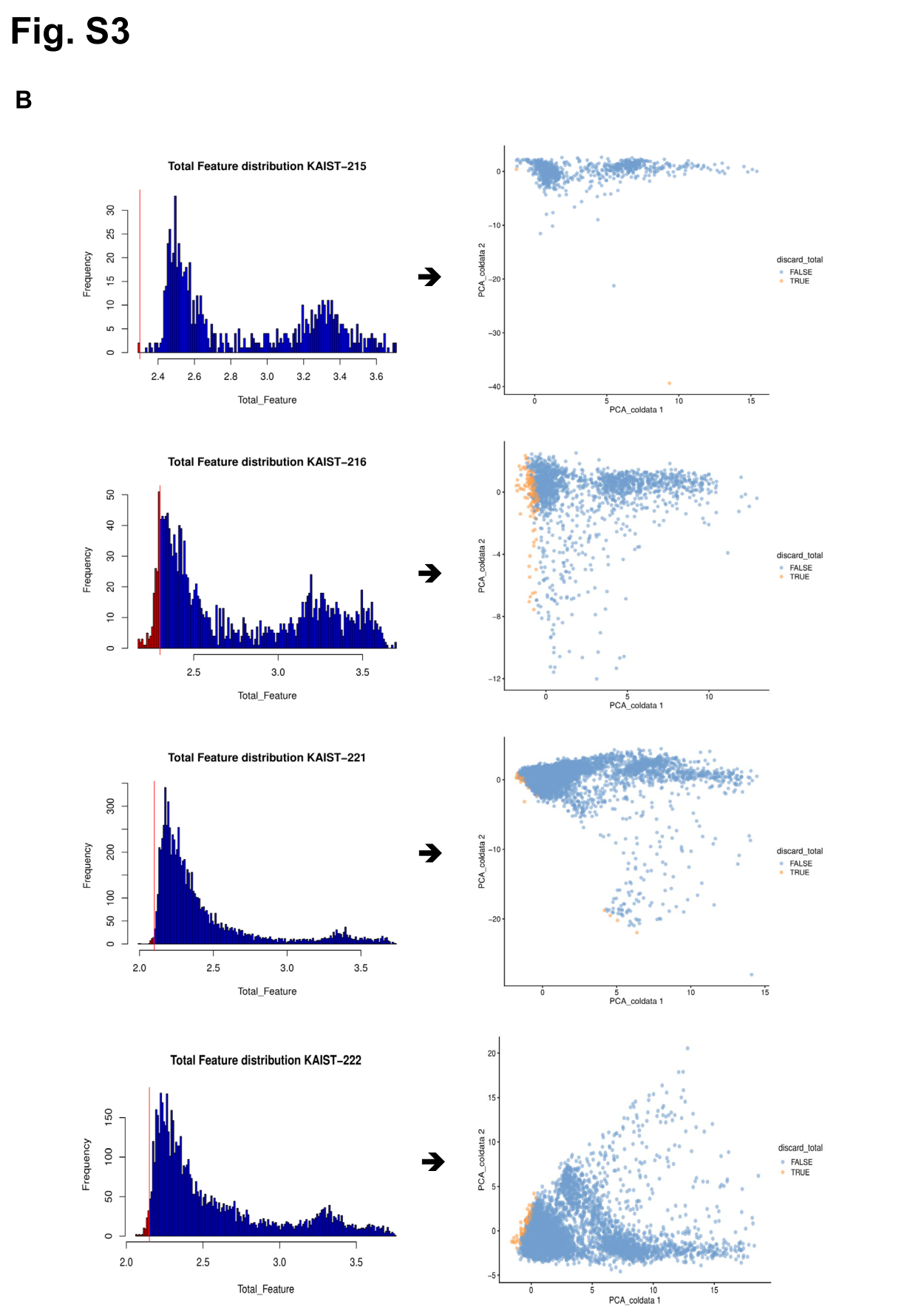

Supplement: Supplementary file 5 [file Image_5.tiff]

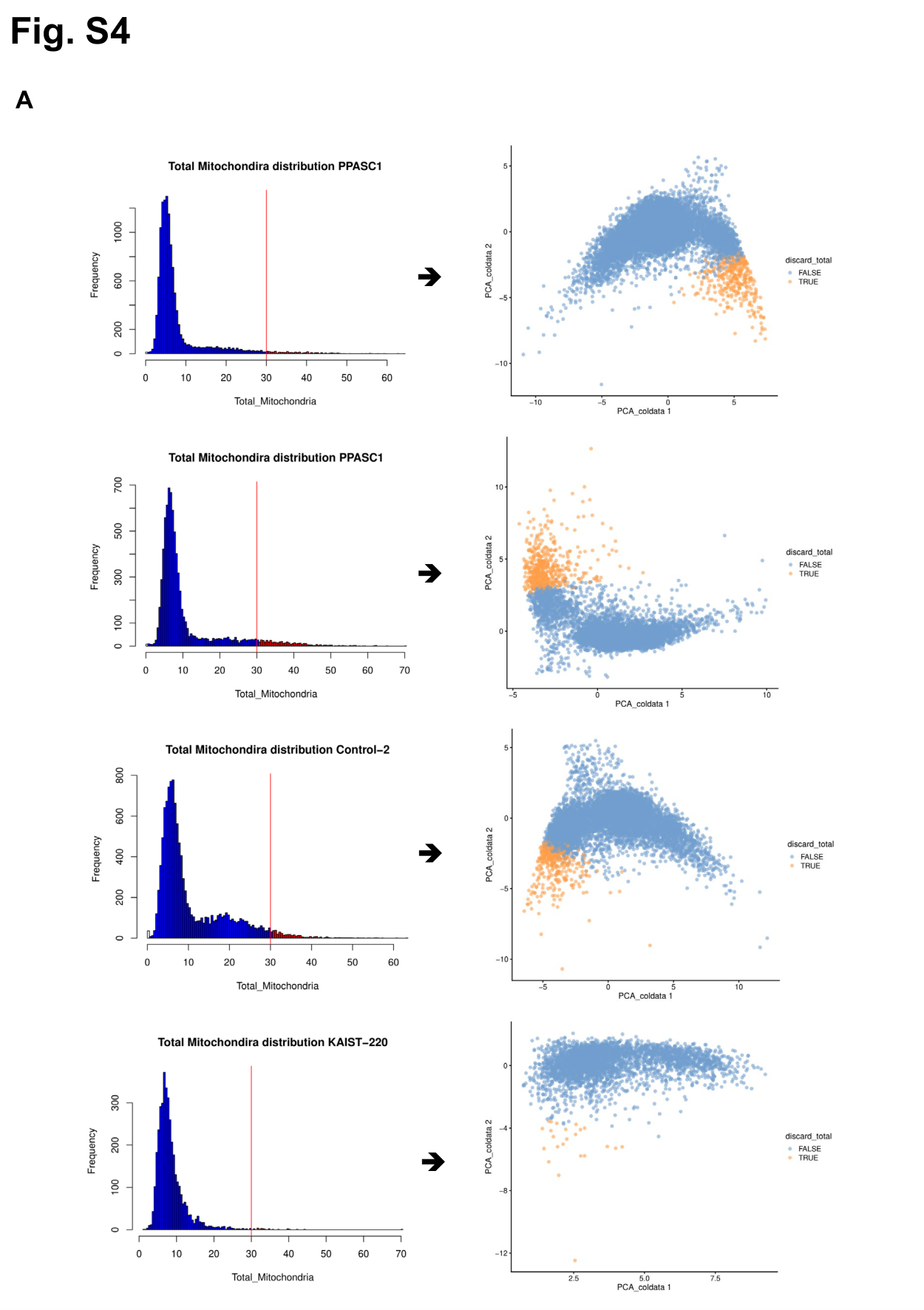

Supplement: Supplementary file 6 [file Image_6.tiff]

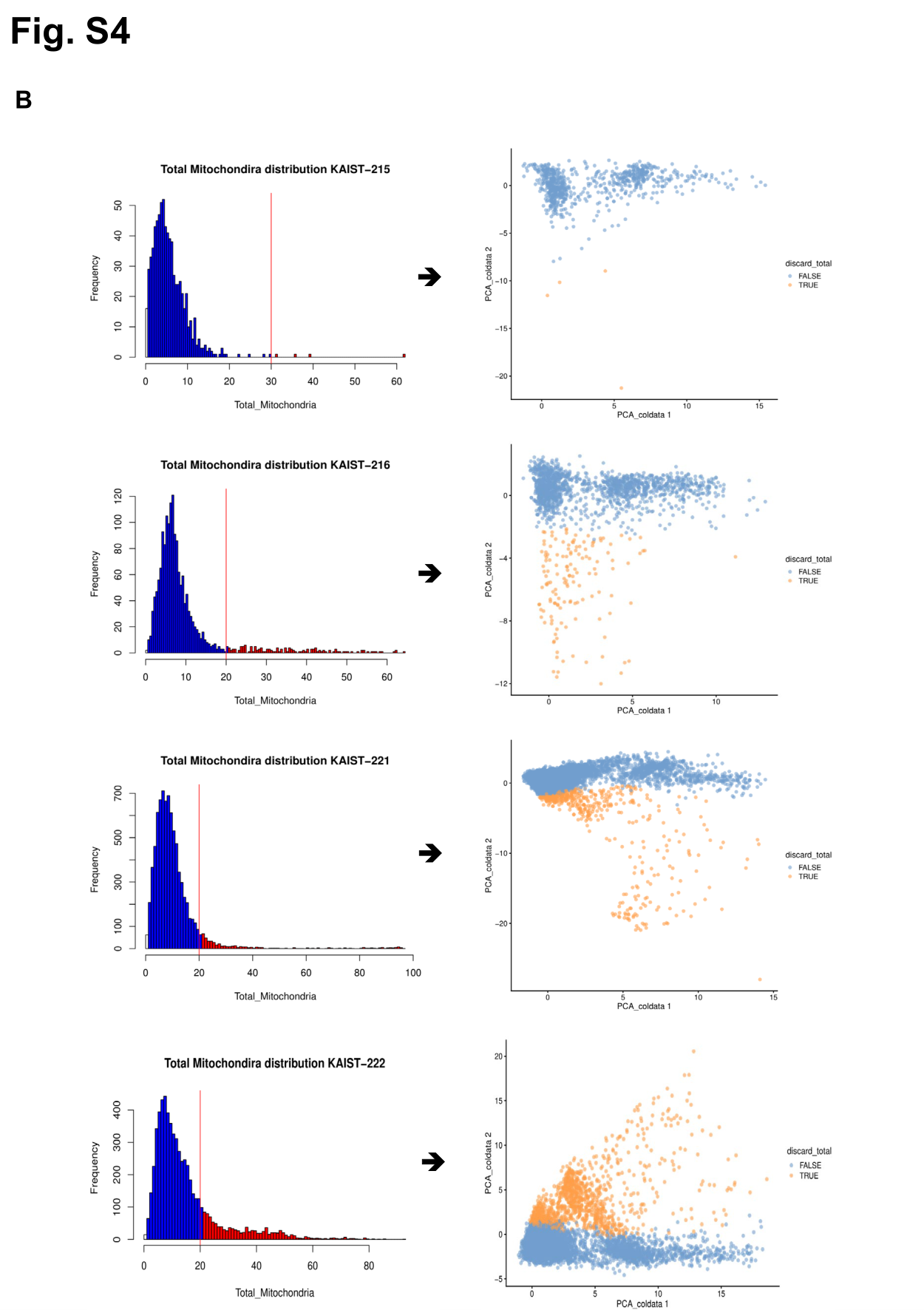

Supplement: Supplementary file 7 [file Image_7.tiff]

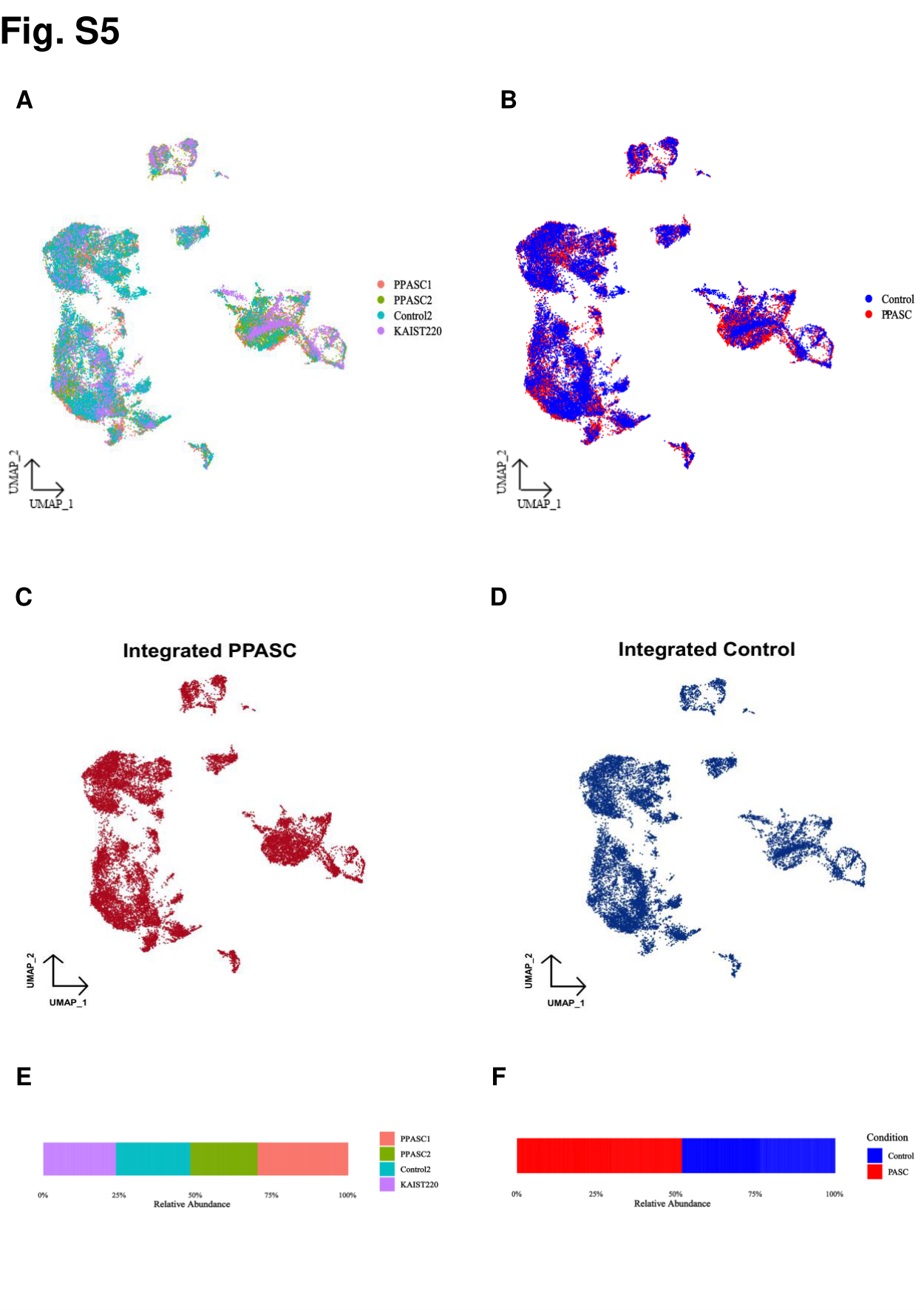

Supplement: Supplementary file 8 [file Image_8.tiff]

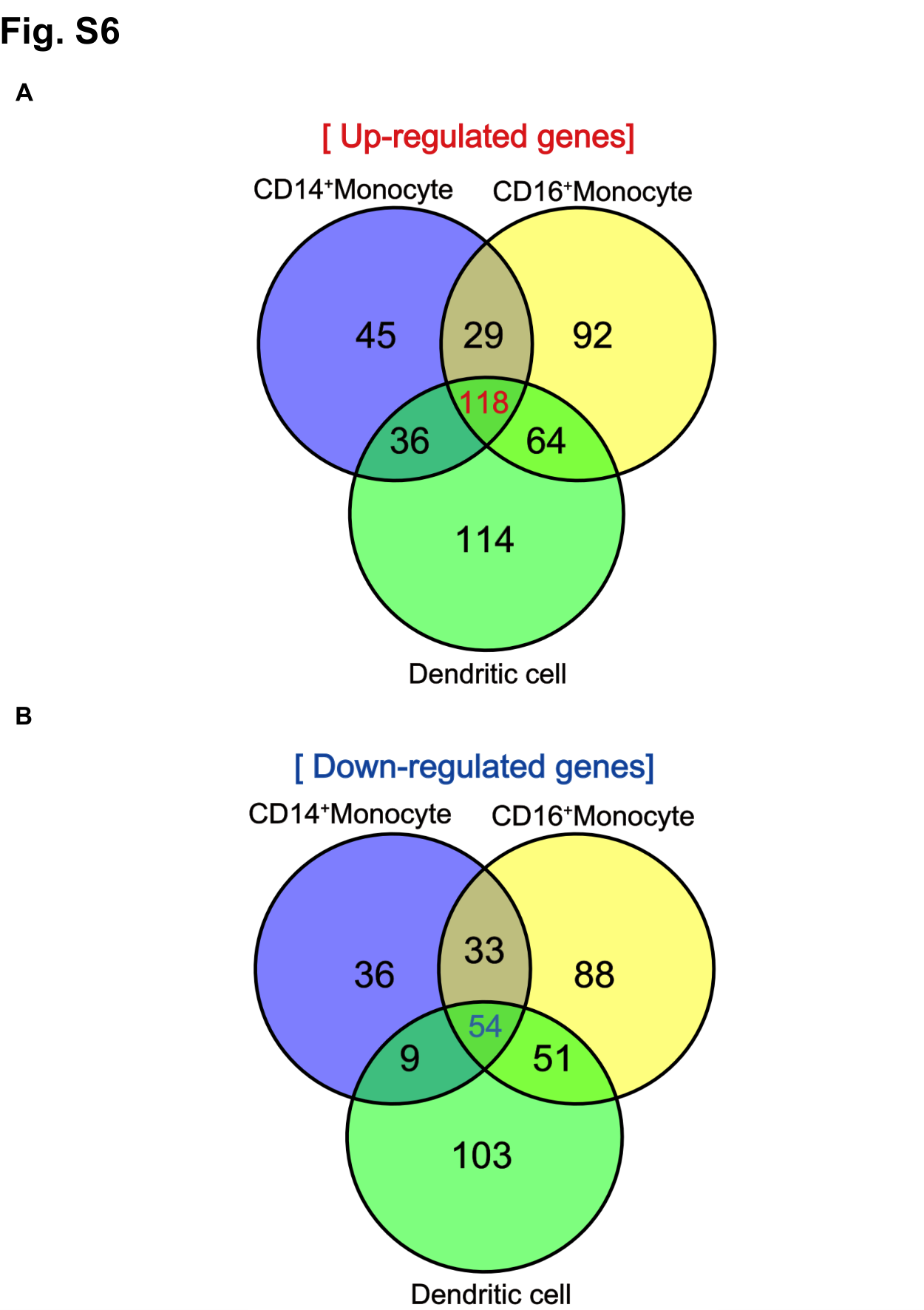

Supplement: Supplementary file 9 [file Image_9.tiff]

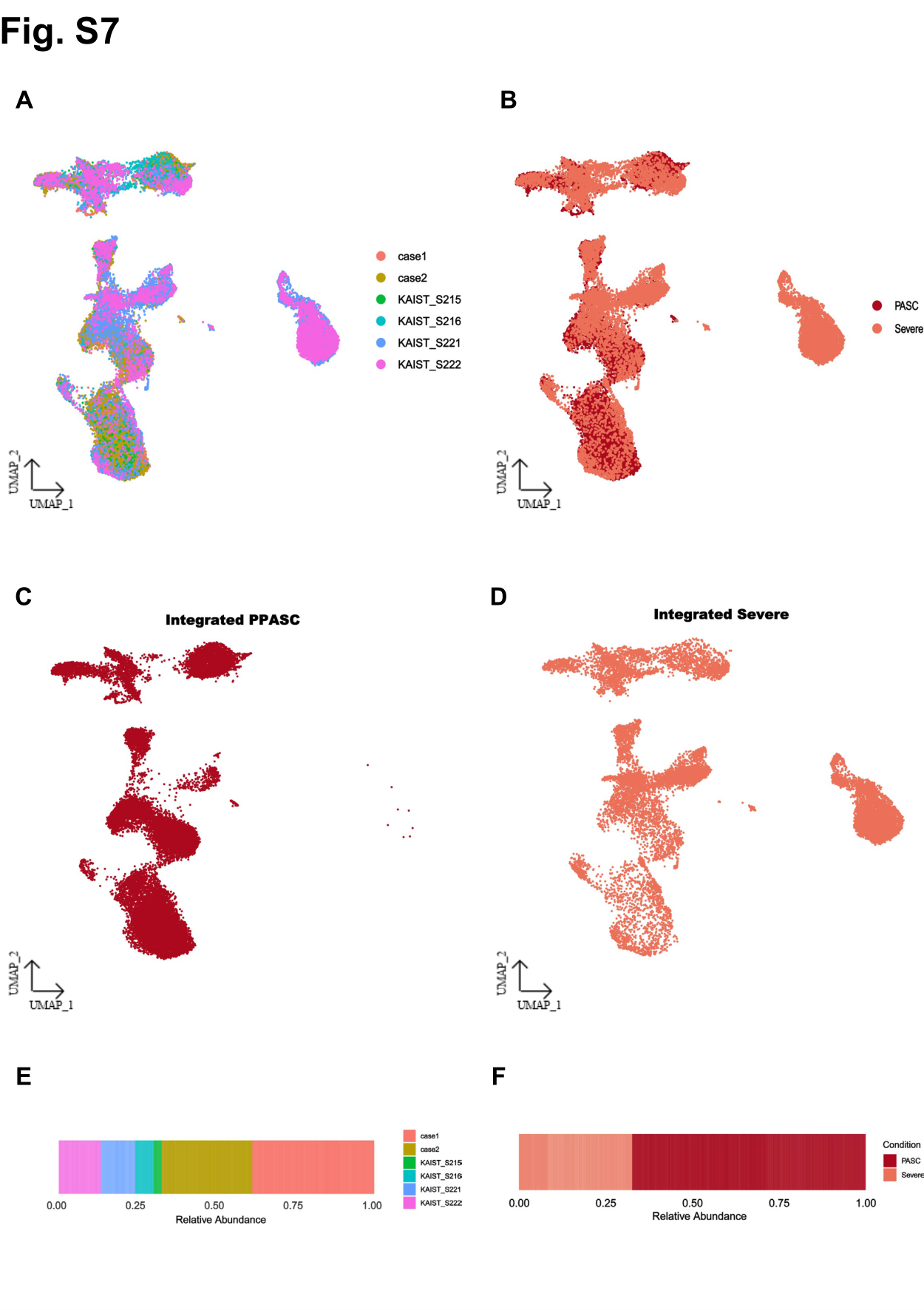

Supplement: Supplementary file 10 [file Image_10.tiff]

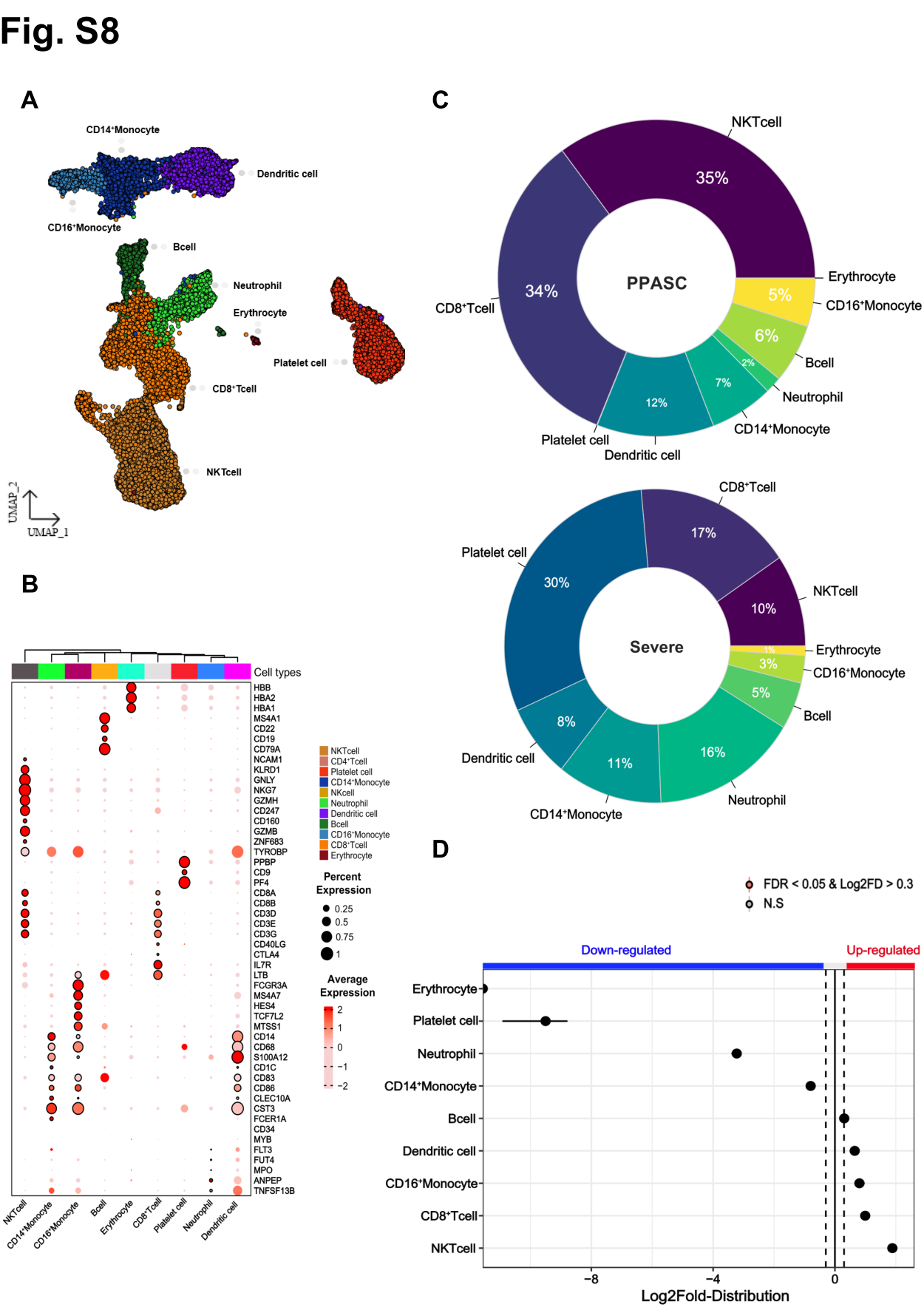

Supplement: Supplementary file 11 [file Image_11.tiff]

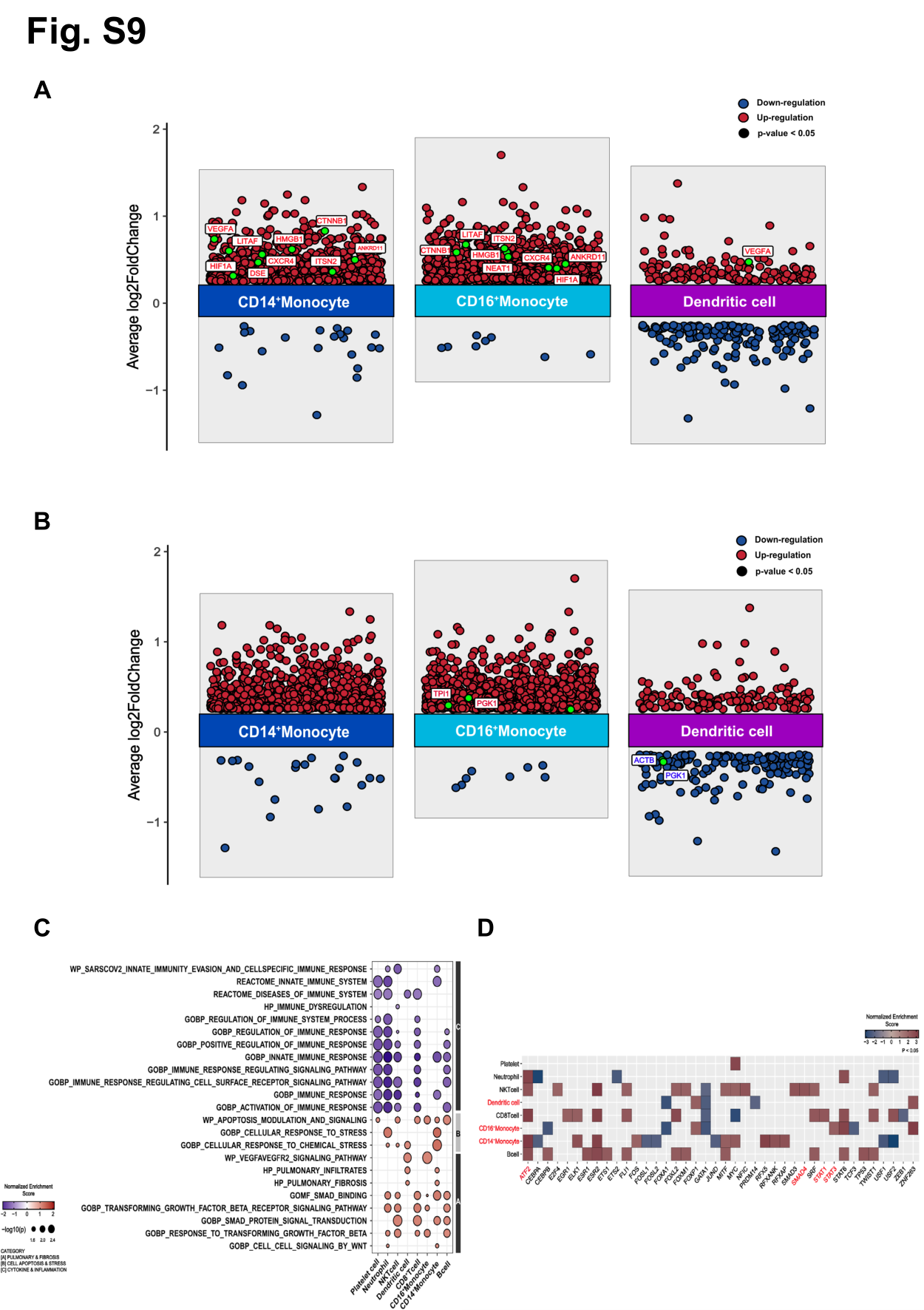

Supplement: Supplementary file 12 [file Image_12.tiff]

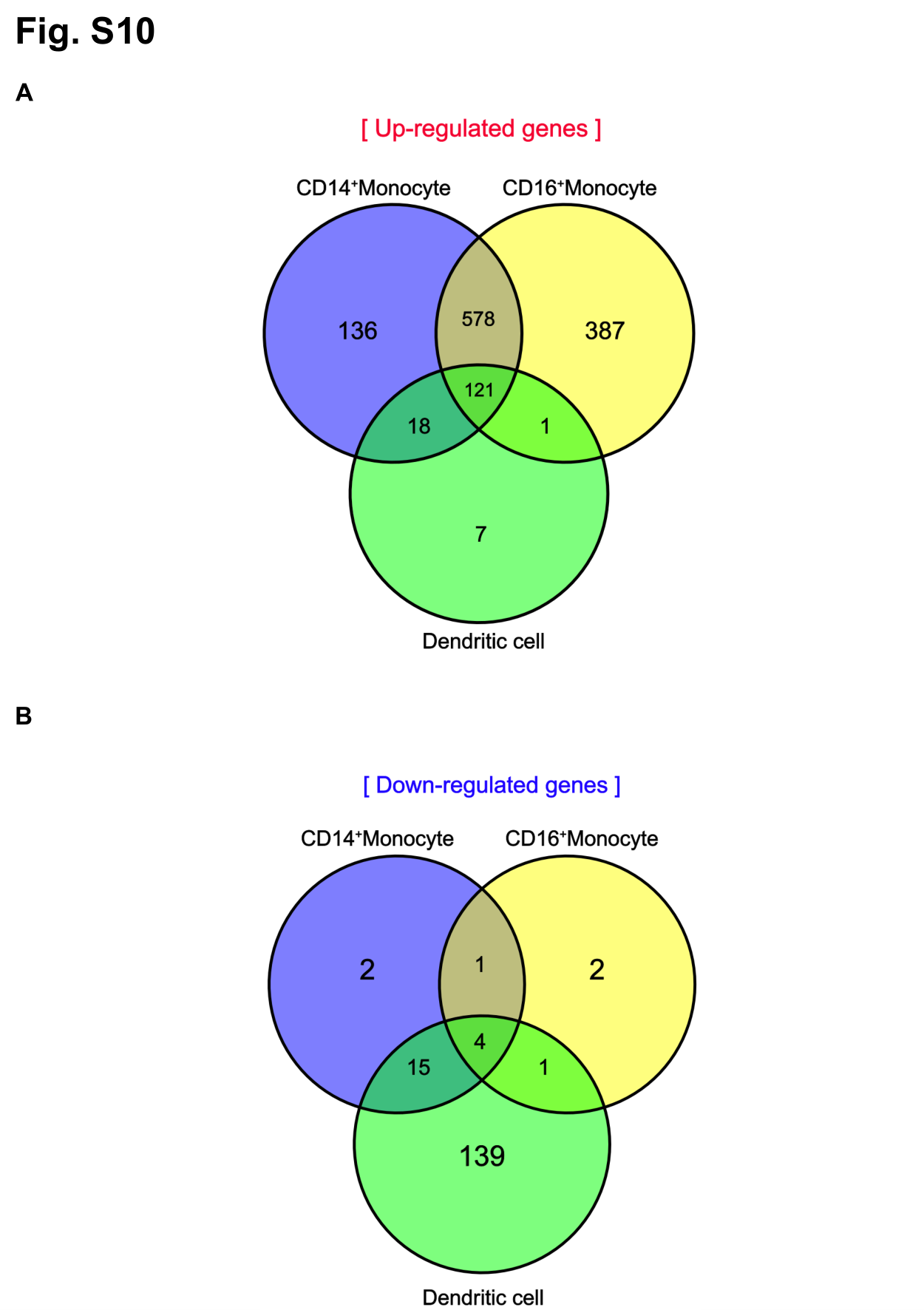

Supplement: Supplementary file 13 [file Image_13.tiff]

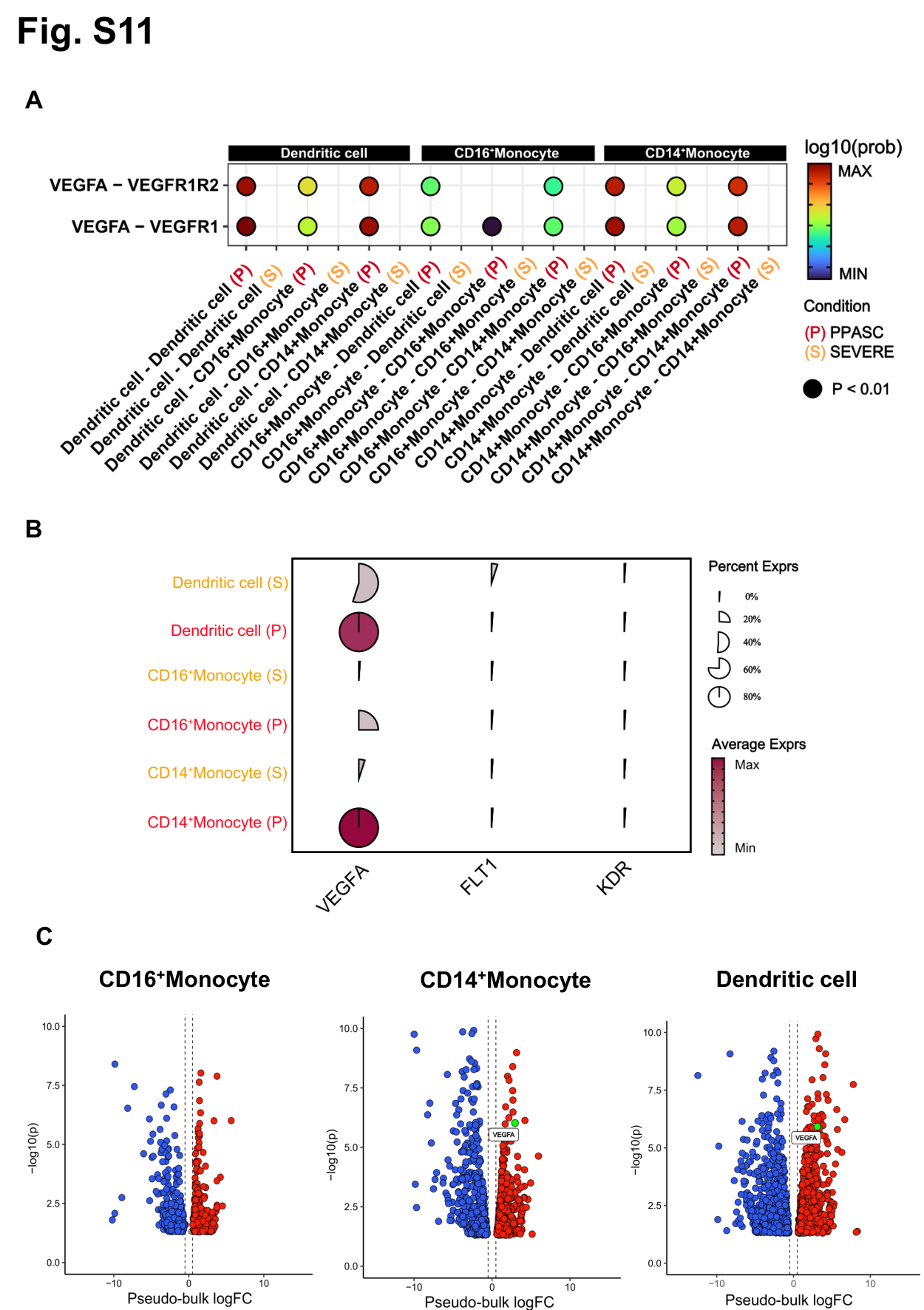

Supplement: Supplementary file 14 [file Image_14.tiff]

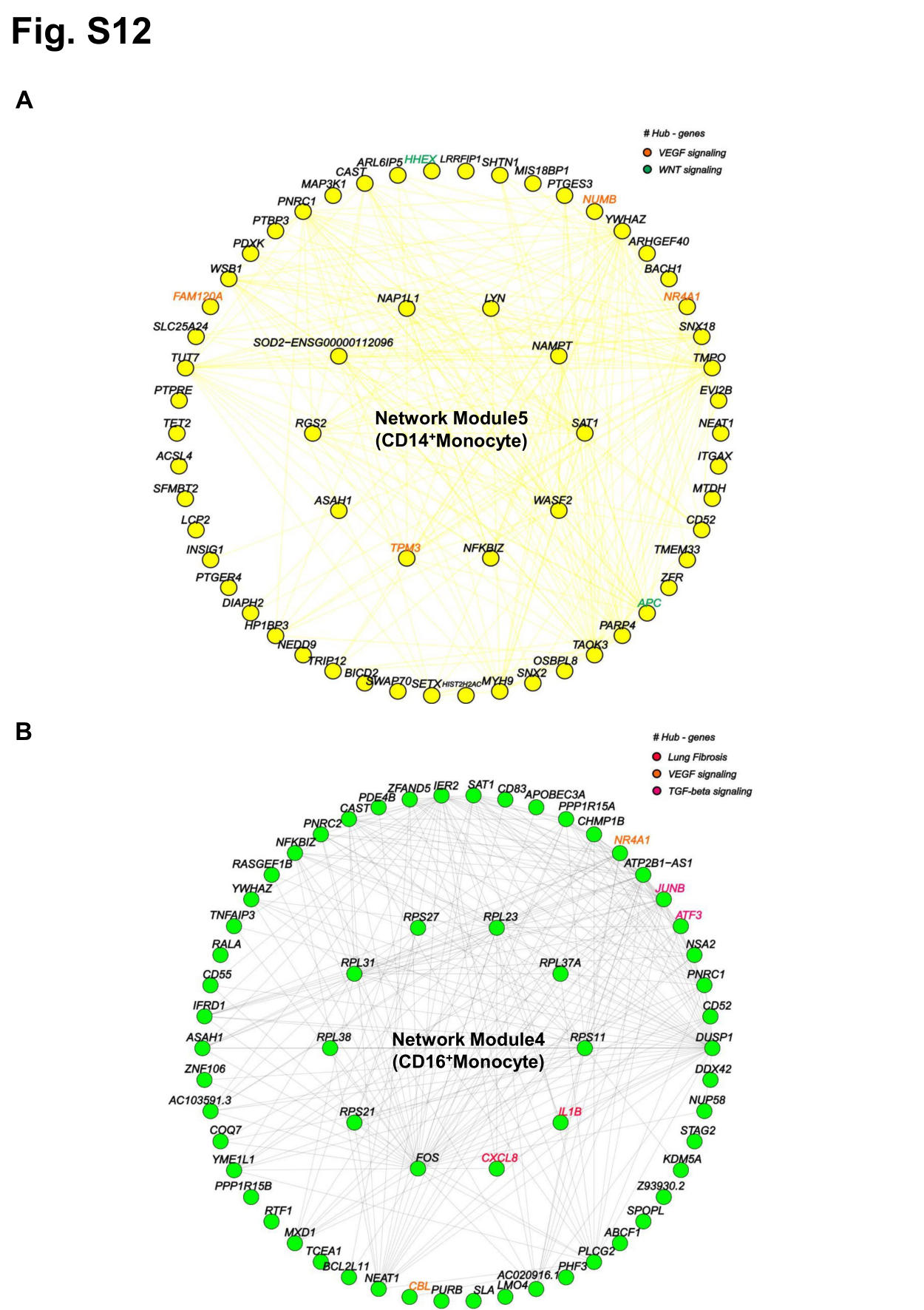

Supplement: Supplementary file 15 [file Image_15.tiff]
