# Supplementary material for: Single‐cell RNA sequencing reveals characteristics of myeloid cells in post-acute sequelae of SARS-CoV-2 patients with persistent respiratory symptoms
Source: Front Immunol. 2024 Jan 8;14:1268510. doi: 10.3389/fimmu.2023.1268510 (PMC10800799; doi:10.3389/fimmu.2023.1268510)
Supplement: Supplementary file 16 [file DataSheet_1.docx]

**Supplementary Figure 1.** **Methods for Empty Droplet Determination. A.** PPASC

(n=2), Control (n=2), and external dataset (n=4) participant’s scRNA-seq reads ranked by Unique

Molecular Identifier (UMI) to remove empty droplets from each sample. Empty droplet cells with

a statistical significance below a False Discovery Rate (FDR) threshold of < 0.01 were

subsequently removed.

**Supplementary Figure 2. UMI Quality Control Droplet Removal. A-B.** Low-quality cells were

identified for PPASC (n=2), Control (n=2), and external dataset (n=4) participants based on the

low of UMI content in each sample, visualized via histogram distribution plots and Principal

Component Analysis (PCA). Cells with low UMI contents or relative outliers in the visualized

PCA were identified and subsequently removed.

**Supplementary Figure 3. Low Quality Cell Removal via Total Feature Distribution. A-B.**

Low-quality cells from for PPASC (n=2), Control (n=2), and the external dataset (n=4) were

identified based on the percentage of gene expression, visualized by PCA, and then removed. Cells

with low gene expression or located as relative outliers in the visualized PCA were marked as

low-quality cells and removed.

**Supplementary Figure 4. Dead Cell Removal via mtDNA Quantity.**

Low-quality cells for PPASC (n=2), Control (n=2), and the external dataset (n=4) were identified based on low mitochondria percent expression (MT) content in each sample and visualized by histogram distribution plots and PCA. Cells with high MT content or relative outliers in the visualized PCA were identified as dead and subsequently removed.

**Supplementary Figure 5. Integration of PPASC and Quality Control Samples.** UMAP

generated using dimensional reduction of the consolidated data, with each sample identified by its

respective sample ID. **B.** UMAP created using dimensional reduction of the integrated data with

samples grouped by their respective group IDs into PPASC (n=2) and Control (n=2) **C.** UMAP

was constructed using dimensional reduction of the integrated data, isolating only the PPASC

samples (n=2). **D.** UMAP was generated using dimensional reduction of the blended data, focusing

exclusively on the Control samples (n=2) **E.** Proportion by sample in the blended data visualized

as a bar stacked plot. **F.** Results of proportion by group in the integrated data, visualized as a bar

stacked plot.

**Supplementary Figure 6. DEGs in PPASC and Control MLC Populations. A.** Venn diagram

illustrates the overlapping up-regulated genes in the total DEG count of MLCs between PPASC

(n=2) and the Control (n=2) group. **B.** Venn diagram displays the overlapping down-

regulated genes in the total DEG count of MLCs between PPASC (n=2) and the Control (n=2)

group.

**Supplementary Figure 7. Integration of PPASC and Severe COVID-19 scRNA-Seq Data. A.**

UMAP was generated using dimensional reduction of the PPASC (n=2) and Severe COVID-19

Data (n=4) data, with each sample denoted its corresponding ID **B.** UMAP was created using

dimensional reduction of the integrated data by PPASC or Severe grouping **C.** UMAP was

constructed using dimensional reduction of the integrated data, isolating only the condition

corresponding to PPASC (n=2). **D.**UMAP generated using dimensional reduction of the Severe

(n=4) blended data. **E.** Proportion of each sample in the blended data, visualized as a bar

stacked plot. **F.** Results of proportion by group in the integrated data, visualized as a bar

stacked plot.

**Supplementary Figure 8: Differential Cell Population Expression in PPASC and Severe**

**Groups. A.** UMAP using dimensional reduction and clustering by cell type. Clusters are

representative of the integrated PPASC (n=2) and Severe (n=4) groups. **B.** Average expression and

percent expression of canonical cell type marker genes from Cell Marker DB 2.0 used to identify

cell type in each cell cluster, visualized as a dot plot. **C**. Calculated proportion of cell types in

the PPASC and Severe groups, visualized as a pie chart. **D.** Permutation and boot strapping

testing for differential cell type proportions between PPASC and Severe groups. The dot plot

illustrates statistically significant relative differences in cell proportions, representing the

Log2 fold distributions (Log2FD) in PPASC compared to Severe.

**Supplementary Figure 9. Differential Gene Expression Patterns in PPASC and Severe**

**group MLCs.** Differentially expressed and upregulated gene with selective labeling of fibrosis-related genes in CD14^+^ and CD16^+^ monocytes, and dendritic cell populations in PPASC (n=2) participants compared to the Severe (n=4) group and visualized as a scatter plot. **B.** Differentially expressed and downregulated genes of glycolysis-related enzymes in CD14^+^ and CD16^+^ monocytes, and dendritic cell populations in PPASC compared to the Severe group, visualized as a scatter plot. Statistically significant up-regulated genes are illustrated via the red dots statistically significant down-regulated genes by blue dots (A-B) **C.** Comparative analysis of functional pathways in PPASC compared to the Severe group across all immune cell types visualized by dot plot and grouped by selective pathway category. Red dots represent up-regulated pathways and blue dots represent down-regulated pathways. **D.** Inferred differentially enriched transcription factors in PPASC compared to the Severe group in whole cell types based on DoRothEA’s target genes database information. Data is visualized as a heatmap with red indicative of up-regulation, blue indicative of down regulation, and transcription factors related fibrosis highlighted in red.

**Supplementary Figure 10. DEGs in MLCs in PPASC and Severe Groups. A.**  The Venn

diagram illustrates the overlapping up-regulated genes from the total DEG count of MLCs

between PPASC (n=2) and the Severe (n=4) group. **B.** The Venn diagram displays the overlapping

down-regulated genes in the total DEG count of MLCs between PPASC and the Severe group.

**Supplementary Figure 11: Cell to Cell Interaction Networks in PPASC and Severe**

**MLCs. A.** Geometric expression in cell-to-cell ligand-receptor interactions and VEGF

signaling interactions calculated between CD14^+^ and CD16^+^ monocytes, and dendritic cells in

PPASC (n=2) and Severe (n=4) groups. Statistically significant and comparable cell-to-cell

interaction geometric expressions are shown on the X-axis, with 'P' and 'S' denoting PPASC and

Severe groups, respectively**. B.** Cell population average and percent expression of VEGF ligand

receptor genes in CD14^+^ and CD16^+^ monocytes, and dendritic cells in PPASC and Severe groups.

Increased expression is denoted by red, and proximity to a circular form signifies a high percent

expression in the cell type cluster. Severe group is denoted in yellow and via the ‘S’ notation, while

the PPASC group is denoted in red and via the ‘P’ notation for each cell population. **C.** For

validation, aggregated single-cell expression data was processed as pseudo-bulk, and differentially

expressed tests were conducted on VEGF ligand genes in PPASC compared to Severe groups. Red

dots signify up-regulation and blue dots signify down-regulation of genes

**Supplementary Figure 12. Upregulated Gene Expression Modules in CD14^+^ and CD16^+^**

**Monocytes in PPASC and Control Groups A.** Correlation network module 5 from CD14^+^ monocytes in PPASC (n=2) compared to Controls (n=2) showcasing the drive hub genes. Genes recognized for their involvement in pathways such as VEGF signaling and WNT signaling are denoted in orange or green lettering, respectively. **B.** Correlation network module 4 from CD16^+^ monocytes in PPASC (n=2) compared to Controls (n=2) showcasing the drive hub genes. Genes recognized for their involvement in pathways such as Lung Fibrosis, VEGF signaling, and TGF- β signaling are denoted in red, orange, and pink lettering, respectively.
