# Supplementary material for: Single‐cell RNA sequencing reveals characteristics of myeloid cells in post-acute sequelae of SARS-CoV-2 patients with persistent respiratory symptoms
Source: Front Immunol. 2024 Jan 8;14:1268510. doi: 10.3389/fimmu.2023.1268510 (PMC10800799; doi:10.3389/fimmu.2023.1268510)
Supplement: Supplementary file 17 [file Table_1.docx]

**Supplementary Table 1. Demographics Table Participants**

|  | **Control 1** | **PPASC 1** | **PPASC 2** |
| --- | --- | --- | --- |
| **Age** | 61 | 52 | 60 |
| **Ethnicity** | Chinese | Japanese | Japanese |
| **Sex** | Female | Male | Female |
| **BMI** | - | 29.3 | 26.2 |
| **History of Asthma** | No | Yes | No |
| **History of COPD** | No | No | Yes |
| **Ever Smoked** | No | No | No |
| **Diabetes Mellitus** | No | No | No |
| **Hypertension** | No | No | No |
| **Heart Disease including CHF and CAD** | Yes | No | Yes |
| **Hospitalized for COVID-19** | No | Yes | Yes |
| **Length of Hospitalization (days)** | - | 60 | 2 |
| **Date Enrolled Post-Acute Infection (months)** | 0 | 9 | 5 |
| **Fully Vaccinated against SARS-CoV-2** | - | Yes | Yes |
| **COVID-19 Symptoms** | - | Shortness of Breath, Fatigue, Vertigo, Headache, Brain Fog, Depression | Chest Pain/Tightness, Neuropathy, Joint Pain |
| **WBC** | - | 5.86 | 10.38 |
| **Monocyte Count** | - | 9.9 | 7.5 |
| **Neutrophil Count** | - | 2.36 | 7.87 |
| **Platelet Count** | - | 264 | 221 |
| **Lymphocyte Count** | - | 2.61 | 1.4 |
| **D-Dimer** | - | 0.41 | 0.48 |
| **CRP** | - | 3.0 | <3.0 |
| **DLCO-c%** | - | 66.4 | 39.2 |

“–” denotes unavailable information.
